# Unraveling the Phylogenetic, Structural, and Functional Dynamics of *CCO* Genes in *Citrus sinensis*, *Olea europaea* var. *sylvestris*, *Populus nigra*, *Prunus dulcis*, and *Punica granatum*: A Comprehensive Bioinformatic Comparative Analysis

**DOI:** 10.3390/genes17080903

**Published:** 2026-07-30

**Authors:** Ummahan Öz

**Affiliations:** Department of Plant and Animal Production, Manisa Celal Bayar University, 45600 Manisa, Türkiye; ummuhan.oz@cbu.edu.tr

**Keywords:** carotenoid cleavage oxygenase, abiotic stress response, gene structure, phylogenetic analysis, cis-acting elements, miRNA

## Abstract

Background/Objectives: *Citrus sinensis*, *Olea europaea* var. *sylvestris*, *Populus nigra*, *Prunus dulcis*, and *Punica granatum* are economically and medicinally important perennial plant species. *Carotenoid cleavage oxygenase (CCO)* genes encode key enzymes involved in carotenoid degradation and play essential roles in plant growth, development, and responses to environmental stresses. In this study, a comprehensive genome-wide comparative analysis of the *CCO* gene family was conducted in *C. sinensis*, *O. europaea* var. *sylvestris*, *P. nigra*, *P. dulcis*, and *P. granatum* to investigate their structural diversity, evolutionary relationships, and potential biological functions. Methods: Chromosomal distribution, phylogenetic relationships, gene structure, conserved protein motifs, homology modeling, subcellular localization, cis-regulatory elements, and miRNA interactions were analyzed. Results: A total of 12, 23, 22, 11, and 17 *CCO* genes were identified in *C. sinensis*, *O. europaea* var. *sylvestris*, *P. nigra*, *P. dulcis*, and *P. granatum*, respectively. Most CCO proteins were acidic, and genes were concentrated on specific chromosomes. Phylogenetic analysis grouped *CCO* genes into three main clades. Gene structure analysis revealed intronless and intron-containing genes of varying lengths. Some CCO proteins possessed all conserved motifs, while others lacked certain motifs or had multiple copies. β-sheets were the predominant secondary structural elements, and CCO proteins were predicted to be localized in chloroplasts, mitochondria, peroxisomes, the cytoplasm, and the nucleus. Stress-related cis-elements and miRNAs were identified. Conclusions: These findings provide valuable insights into the diversity and evolutionary characteristics of the *CCO* gene family and suggest that *CCO* genes may contribute to plant stress responses and metabolic processes. Overall, this study provides a comprehensive comparative analysis of the *CCO* gene family in these five perennial plant species and offers a valuable genomic resource for future functional characterization, comparative genomic studies, and molecular breeding applications.

## 1. Introduction

Carotenoids in plants are produced from isopentenyl diphosphate and dimethylallyl diphosphate via the 2-C-methyl-D-erythritol-4-phosphate pathway, with apocarotenoids formed through their cleavage playing essential roles in growth, development, and stress responses [1,2]. Carotenoid cleavage oxygenases (CCOs), which break the conjugated double bond in the carotenoid polyene chain to produce various apocarotenoids, play an essential role in physiological processes such as phytohormone production, plant growth and development, and responses to abiotic and biotic stresses [3,4]. The *CCO* gene family has been identified in numerous plants, and based on substrate differences, it is divided into two subfamilies: *9-cis-epoxycarotenoid cleavage dioxygenase (NCED)* and *carotenoid cleavage dioxygenase (CCD)* [4]. Various studies have reported on *NCED* genes, such as the activation of seed dormancy in *Arabidopsis* through the combined activity of *AtNCED5*, *AtNCED6*, and *AtNCED9*, the inhibition of seed germination by *AtNCED2*, *AtNCED5*, and *AtNCED9* through increased abscisic acid levels, the role of *AtNCED1* in the response to water stress, the induction of *OsNCED3*, *OsNCED4*, and *OsNCED5* genes under salt and abscisic acid treatments, and the involvement of the *LeNCED1* gene in drought tolerance in tomato [5]. In addition to these, it has been stated that *CCD* genes play a role in important physiological processes, such as the formation of apocarotenoid volatile compounds in certain fruits and flowers, fruit development, abiotic stress response, and the synthesis of strigolactin, a hormone associated with reproductive development and shoot branching [6].

Studies on *CCO* genes have been conducted in species such as *Saccharum* spp. R570 cultivar L., Huayao Orah mandarin, *Malus domestica* (Suckow) Borkh., *Durio zibethinus* L., *Liriodendron chinense* (Hemsl.) Sarg., *Brassica rapa* L., *Brassica oleracea* L., *Coffea arabica* L., *Arachis hypogaea* L., *Cucumis sativus* L., *Gossypium raimondii* Ulbr., *G. arboreum* L., *G. hirsutum* L., *Helianthus annuus* L., *Glycine max* (L.) Merr., *Betula platyphylla* Sukaczev, *Litchi chinensis* Sonn., *Pyrus bretschneideri* Rehder, *Fragaria vesca* L., *Prunus mume* (Siebold) Siebold & Zucc., and *Prunus persica* (L.) Batsch [3,4,6,7,8,9,10,11,12,13,14,15,16,17,18]. However, a comprehensive genome-wide analysis has not been reported in *Citrus sinensis* (L.) Osbeck, *Olea europaea* var. *sylvestris* (Mill.) Lehr, *Populus nigra* L., *Prunus dulcis* (Mill.) D. A. Webb, and *Punica granatum* L., according to the literature review.

*C. sinensis*, belonging to the Rutaceae family, is an economically significant plant containing various volatile compounds such as limonene, *α*-terpinene, ϒ-terpinene, *α*-pinene, *β*-pinene, *α*-terpineol, *β*-terpineol, geranial, *β*-geraniol, neral, myrcene, valencene, linalool, (3R)-(-)-linalool, *α*-caryophyllene, 1-octanol, nerol, and sabinene; flavonoids such as hesperetin, hesperidin, narirutin, tangeretin, limocitrin, naringin, naringenin, sakuratin, and limocitrol; steroids such as *β*-sitosterol and *β*-sitosterol-3-O-*β*-D-glucopyranoside; coumarins such as isopimpinellin, limettin, scoparone, bergapten, osthol, and bergaptol; and vitamins A, B1, B2, B3, B5, B6, C, D, E, and K [19]. Due to its bioactive components, it demonstrates therapeutic effects against conditions such as diarrhea, constipation, colic, cramps, cough, bronchitis, the common cold, menstrual disorders, depression, anxiety, and hypertension [20].

*Olea europaea* L., a member of the Oleaceae family, has two varieties: *Olea europaea* subsp. *europaea* var. *europaea* (cultivated olive) and *Olea europaea* subsp. *europaea* var. *sylvestris* (oleaster) [21]. Oils obtained from both cultivated and wild olives have significant effects on human health, and it has been reported that the antioxidant activity of oils derived from wild olives is equal to or higher than that of cultivated forms [22]. Furthermore, wild olives are considered a valuable natural genetic resource due to their high resistance to various environmental, climatic, and disease conditions, making the investigation of their genetic characteristics beneficial [22].

*Populus nigra* is a plant belonging to the Salicaceae family, distributed from Northern Europe to Central Asia and the coasts of North Africa, and is medicinally valuable due to its secondary metabolites such as phenolic compounds and terpenes [23]. *P. nigra* contains important flavonoids such as apigenin, chrysin, pinobanksin, pinocembrin, pinostrobin, kaempferol, and quercetin, which possess a wide range of therapeutic effects, including anti-inflammatory, antioxidant, antimetastatic, antiangiogenic, anti-diabetic, antihypertensive, antibacterial, antiviral, antimalarial, antiparasitic, antiprotozoal, antitrypanosomal, antiobesity, antiallergic, antiatherosclerotic, cardioprotective, nephroprotective, neuroprotective, hepatoprotective, vasodilator, antiulcer, antivenom, anxiolytic, analgesic, estrogenic/antiestrogenic, antiischemic, and antidepressant properties [24].

*Prunus dulcis*, belonging to the Rosaceae family, is an economically and medicinally significant plant cultivated in over 50 countries, with 95% of production coming from the Mediterranean Basin, Australia, and California, and it contains important fatty acids such as linoleic acid, palmitic acid, palmitoleic acid, and stearic acid, as well as secondary metabolites like lignans, hydroxycinnamic acid, sinapic acid, hydrobenzoic acid, catechin, epicatechin, stilbene, and terpenoids [25,26]. *P. dulcis* is not only known for its anticarcinogenic, antioxidant, anti-inflammatory, anti-atherogenic, and hepatoprotective effects, but also for its ability to reduce blood pressure, cholesterol levels, and the risk of obesity-related disorders [27].

*Punica granatum*, a member of the Lythraceae family, contains high levels of vitamin C, iron, calcium, phosphorus, 17 types of amino acids, alkaloids, flavonoids, tannins, and organic acids [28]. Due to its significant compounds, it possesses anti-inflammatory, anti-depressant, anti-cancer, cardio-protective, antioxidant, anti-obesity, anti-diarrheal, anti-diabetic, antimalarial, and anti-fibrotic properties, and it also contributes to improving male fertility [29]. In addition, it is mentioned to be used in traditional Chinese medicine for the treatment of atherosclerosis, hyperlipidemia, peptic ulcers, various types of cancer, hypertension, and oral diseases, in the Ayurvedic system for treating dysentery and diarrhea, and in the Greco-Arabic medical system for the treatment of chest pain, bile disorders, cough, and jaundice [28].

Secondary metabolites, which contribute to the medicinal properties of plants, not only provide characteristics such as color, scent, and taste but also play a role in activating defense mechanisms against abiotic and biotic stress conditions, with their production increasing under stress [30]. It is stated at the beginning of the article that *CCO* genes play a role in the stress mechanism. The selection of *Citrus sinensis*, *Olea europaea* var. *sylvestris*, *Populus nigra*, *Prunus dulcis*, and *Punica granatum* in this study is based on two reasons: first, *CCO* genes have not been previously studied in these species, and second, this allows for a comparison of *CCO* genes across plant samples from different families. Furthermore, these five species are known to possess significant medicinal properties. This study aims to conduct a comprehensive analysis of *CCO* genes in *C. sinensis*, *O. europaea* var. *sylvestris*, *P. nigra*, *P. dulcis*, and *P. granatum*. By selecting these species from different families, the study seeks to investigate the structural and functional diversity of *CCO* genes and to determine their potential roles in stress-related processes. Within this scope, various aspects of *CCO* genes have been evaluated, including their identification, physicochemical properties, chromosomal distribution, phylogenetic relationships, gene structure, conserved protein motifs, homology modeling, protein subcellular localization, cis-regulatory elements, and miRNA interactions.

Unlike previous studies focusing on individual species, the present study provides a comprehensive comparative analysis of the *CCO* gene family across five phylogenetically distinct woody species. This comparative approach reveals conserved and species-specific differences in gene numbers, gene structure, conserved motifs, regulatory elements, and predicted functional characteristics, thereby enhancing our understanding of the diversity and evolutionary patterns of the *CCO* gene family and providing valuable insights for future functional and evolutionary studies.

## 2. Materials and Methods

The materials of the article consist of *Citrus sinensis*, *Olea europaea* var. *sylvestris*, *Populus nigra*, *Prunus dulcis*, and *Punica granatum*.

### 2.1. Identification and Physicochemical Analysis of CCO Genes in Citrus sinensis, Olea europaea var. sylvestris, Populus nigra, Prunus dulcis, and Punica granatum

The protein sequences for each species were initially downloaded from the NCBI (National Center for Biotechnology Information) database and subsequently analyzed using the BLASTP tool (Protein-basic local alignment search tool) in QIAGEN CLC Genomics Workbench 24.0.1. Therefore, the downloaded sequences were compared with the unique protein sequences of each species. After installing the QIAGEN CLC Genomics Workbench 24.0.1 program, Pfam-A V36 was downloaded into the system. Using this database, a PFAM Domain Search was performed. Upon completion of the process, PFAM ID (PF03055) was entered, and the filtering option was selected. The obtained data were then copied and pasted into Microsoft Excel. Subsequently, repetitive sequences were eliminated, and potentially promising CCO proteins were identified. Following this process, the protein, genome, and CDSs of the identified *CCO* genes were downloaded from NCBI. Each was separately filed, and each species was sorted by chromosome number, with genes renamed accordingly. For this naming, “CCO” was added after the initial letters of the plant’s Latin name, followed by numbering (e.g., *CsCCO1*). After naming the genes for each species, a table was created. The physical position section of the table was filled with data obtained from NCBI, while the physicochemical section was completed using the Expasy ProtParam program (Swiss Institute of Bioinformatics, Lausanne, Switzerland) [31].

### 2.2. Chromosomal Distribution

The chromosomal locations of each gene were obtained from NCBI, and a table was created. These data were transferred to the template file in the MG2C (MapGene2Chromosome) v2.1 software [32] and the chromosomal positions of each gene were visualized.

### 2.3. Phylogenetic Analysis

To perform phylogenetic analysis, the amino acid sequences of *C. sinensis*, *O. europaea* var. *sylvestris*, *P. nigra*, *P. dulcis*, and *P. granatum* were first uploaded to the MEGA 11 software [33]. Alignment was carried out using the MUSCLE algorithm available in the program. The aligned data were exported in MEGA format, and these data were subsequently used to construct a phylogenetic tree. The Maximum Likelihood Tree method was applied, utilizing the Jones–Taylor–Thornton (JTT) substitution model and bootstrap analysis with 1000 replicates. After constructing the phylogenetic tree, it was downloaded to the computer in Newick format. The file was then uploaded to the Interactive Tree of Life (iTOL) v6.7.3 software [34] for visualization. Finally, after completing all necessary adjustments, the tree was downloaded in PNG format and prepared as a Figure.

### 2.4. Gene Structure and Conserved Protein Motif Analysis

Exon-intron regions were identified using the Gene Structure Display Server (GSDS 2.0) [35], which compared genomic sequences to their predicted coding sequences (CDS). To analyze CCO protein sequences, the MEME (Multiple Em for Motif Elicitation) Suite version 5.5.1 [36] was utilized. Motif details were further examined using the MAST (Motif Alignment & Search Tool). The analysis was conducted in classical mode, with parameters set to a maximum of 10 motifs and an optimal width range of 6 to 50.

### 2.5. Homology Modeling of CCO Proteins

The intensive mode of the Phyre2 (Protein Homology/Analog Recognition Engine V 2.0) program [37] was used to perform homology modeling of CCO proteins. This program utilizes sophisticated remote homology detection algorithms to construct 3D protein models and predict ligand binding sites. The modeling workflow includes several stages: detecting homologous sequences, scanning the fold database, performing loop modeling, and positioning side chains to complete the structure.

### 2.6. Protein Subcellular Localization Analysis

Subcellular location analysis of CCO proteins from each species was conducted using the WoLF PSORT software [38]. Initially, the amino acid sequences were uploaded into the software. Upon completion of the analysis, the predicted subcellular locations and corresponding numerical values were saved in a Microsoft Excel file. This data was then used to generate a heatmap with the ClustVis: a web-based platform for visualizing clustering of multivariate data (BETA) [39]. The information from Excel was transferred into the tool, and the heatmap was generated.

### 2.7. Cis-Acting Elements Analysis

The promoter sequences (2 Kb upstream of the start codons) of the *CCO* genes were retrieved from the NCBI database, analyzed for cis-regulatory elements using the PlantCARE database [40], and the identified elements were counted with Microsoft Excel. The obtained data were then transferred to ClustVis software [39], and a heatmap was generated.

### 2.8. miRNA Analysis

The coding sequences (CDS) of CCOs were uploaded to the psRNATarget database [41] to identify potential target miRNAs. The resulting data were copied into Microsoft Excel, each miRNA was searched in the microRNA database (miRBase) [42], and plant-specific miRNAs were selected.

## 3. Results

### 3.1. Identification and Physicochemical Analysis of CCO Genes in C. sinensis, O. europaea var. sylvestris, P. nigra, P. dulcis, and P. granatum

Based on the analysis conducted using protein sequences for each species, 12 *CCO* genes were identified in *C. sinensis*, 23 in *O. europaea* var. *sylvestris*, 22 in *P. nigra*, 11 in *P. dulcis*, and 17 in *P. granatum*. The analysis revealed that the molecular weights of *CsCCO* genes ranged from 55,169.40 Da to 73,877.83 Da, with protein lengths varying between 485 and 656 amino acids. Similarly, the molecular weights of *O. europaea* var. *sylvestris CCO* genes ranged from 13,642.40 Da to 71,373.93 Da, with protein lengths varying between 124 and 628 amino acids. For *P. nigra*, the molecular weights of *CCO* genes ranged from 50,011.44 Da to 70,536.05 Da, and their protein lengths varied between 440 and 625 amino acids. The molecular weights of *P. dulcis CCO* genes ranged from 52,984.84 Da to 70,384.87 Da, with protein lengths varying between 469 and 632 amino acids. Finally, for *P. granatum*, the molecular weights of *CCO* genes ranged from 11,280.71 Da to 72,731.22 Da, while the protein lengths ranged from 101 to 653 amino acids (Supplementary Table S1).

It was observed that the theoretical isoelectric points (pIs) of CsCCO proteins ranged from 5.48 to 8.84, while the pIs of OeCCO proteins ranged from 4.81 to 8.95. In both cases, the majority of the proteins were acidic (pI < 7). It was determined that the pIs of PnCCO ranged from 5.41 to 9.01, with the majority being acidic. The pIs of PdCCO proteins were found to range from 5.37 to 6.97, with all of them exhibiting an acidic character. Finally, it was analyzed that the pIs of PgCCO proteins ranged from 5.68 to 8.54, with the majority being acidic (Supplementary Table S1).

### 3.2. Chromosomal Distribution

**Figure 1:**
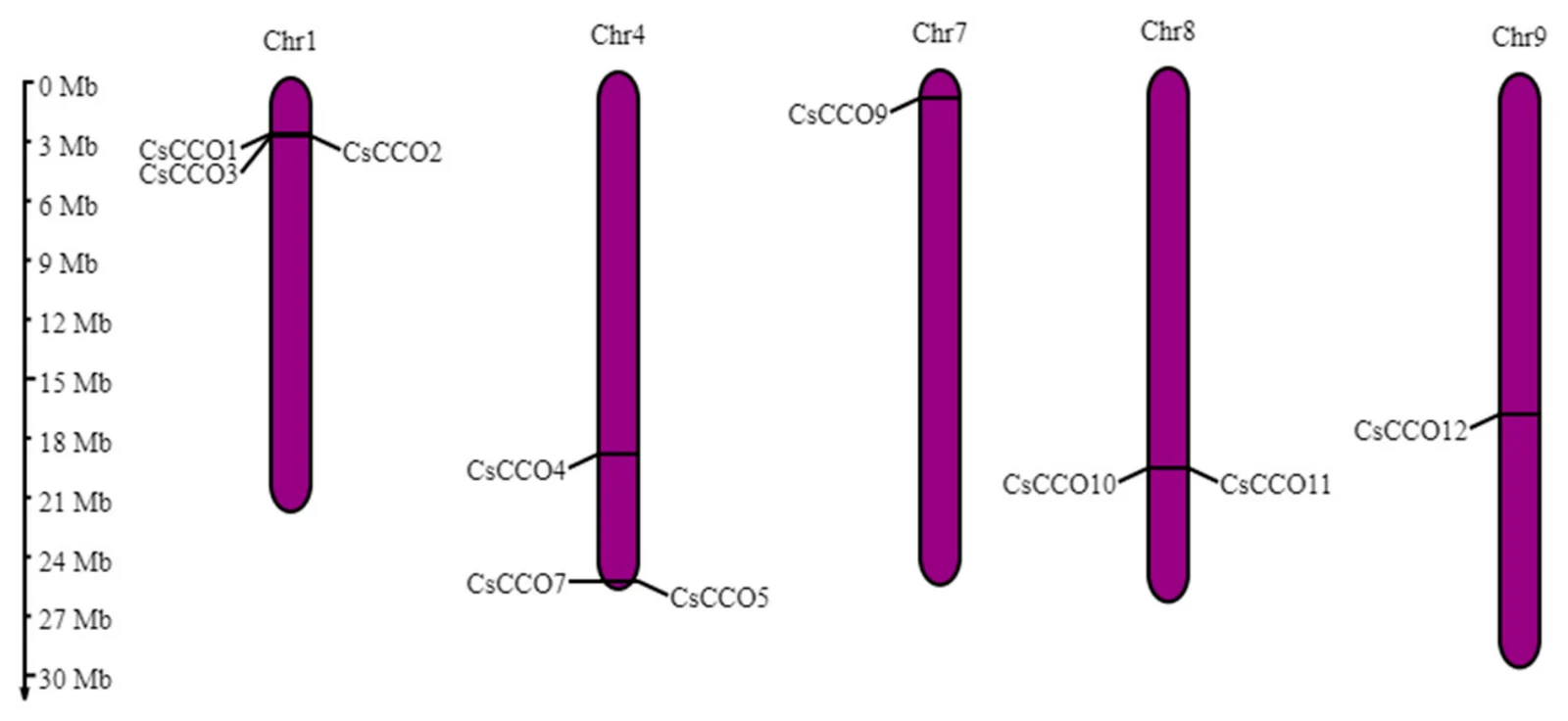
Chromosomal distribution of *CsCCO* genes in *C. sinensis.* The vertical axis indicates chromosome length in megabases (Mb).

*C. sinensis* possesses 9 chromosomes, with *CCO* genes identified only on chromosomes 1, 4, 7, 8, and 9. The fewest *CCO* genes were found on chromosome 9, while the highest number of genes was observed on chromosome 4 (Figure 1). The absence of *CsCCO6* and *CsCCO8* genes in the figure is due to the overlapping start positions of *CsCCO6* with *CsCCO7* and *CsCCO8* with *CsCCO9* (Supplementary Table S1).

**Figure 2:**
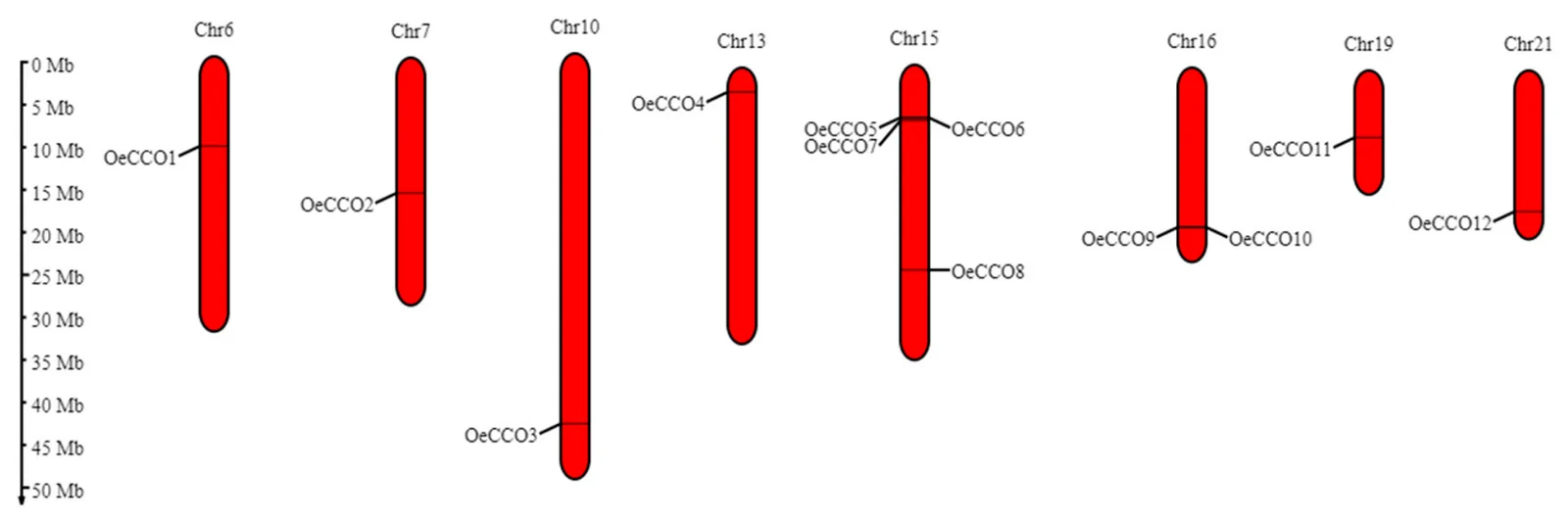
Chromosomal distribution of *OeCCO* genes in *O. europaea* var. *sylvestris.* The vertical axis indicates chromosome length in megabases (Mb).

*O. europaea* var. *sylvestris* has 23 chromosomes, with *CCO* genes observed only on chromosomes 6, 7, 10, 13, 15, 16, 19, and 21. The chromosome containing the highest number of genes is chromosome 15 (Figure 2). The region between *OeCCO13* and *OeCCO23* is a scaffold.

**Figure 3:**
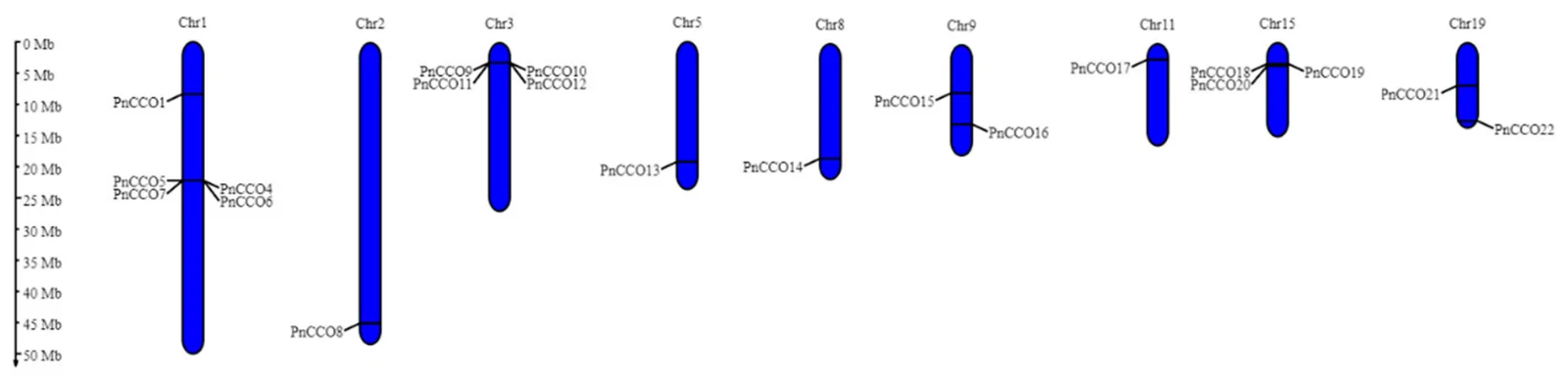
Chromosomal distribution of *PnCCO* genes in *P. nigra.* The vertical axis indicates chromosome length in megabases (Mb).

*P. nigra* has 19 chromosomes, with *CCO* genes detected only on chromosomes 1, 2, 3, 5, 8, 9, 11, 15, and 19. Chromosome 1 contains the highest number of *CCO* genes, while chromosomes 2, 5, 8, and 11 each have only one gene (Figure 3). The absence of *PnCCO2* and *PnCCO3* in the figure is due to their overlapping start positions with *PnCCO4* (Supplementary Table S1).

**Figure 4:**
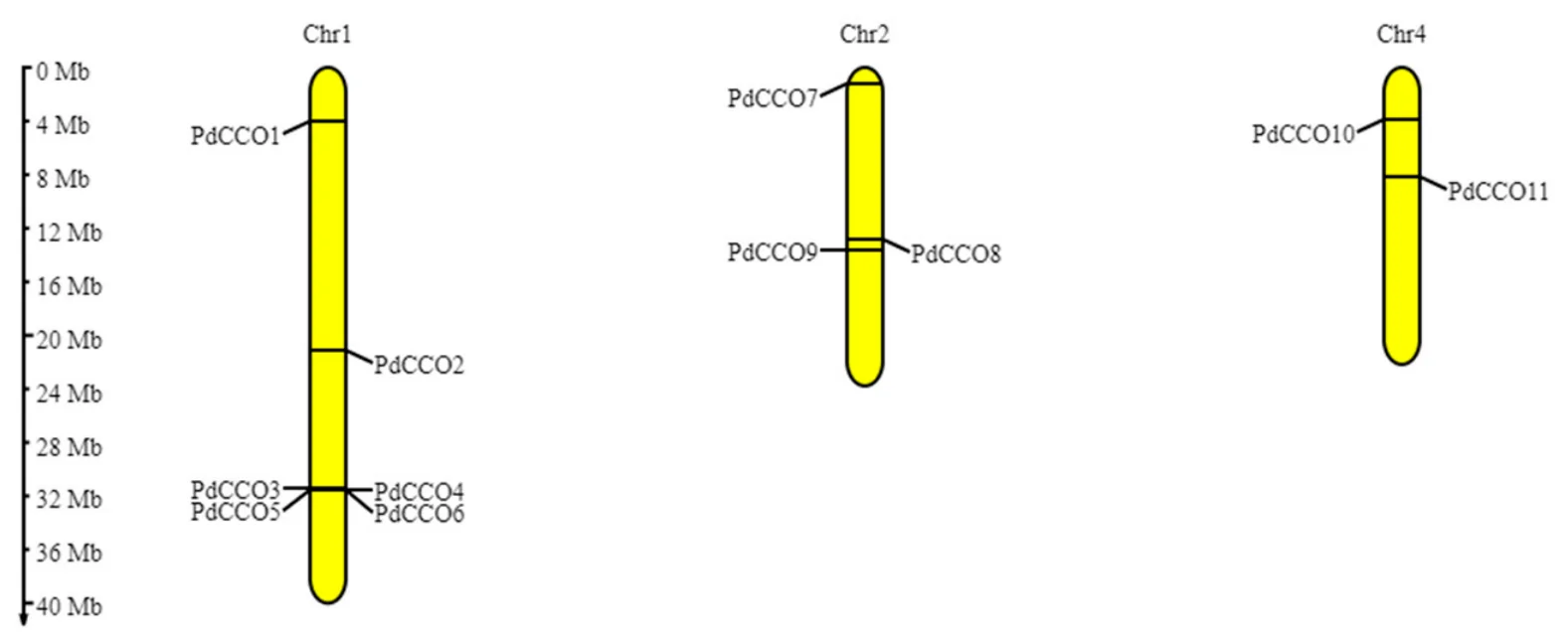
Chromosomal distribution of *PdCCO* genes in *P. dulcis.* The vertical axis indicates chromosome length in megabases (Mb).

*P. dulcis* has 8 chromosomes, with *CCO* genes located on chromosomes 1, 2, and 4. The analysis shows that chromosome 1 holds the highest number of genes, while chromosome 4 contains the fewest (Figure 4).

**Figure 5:**
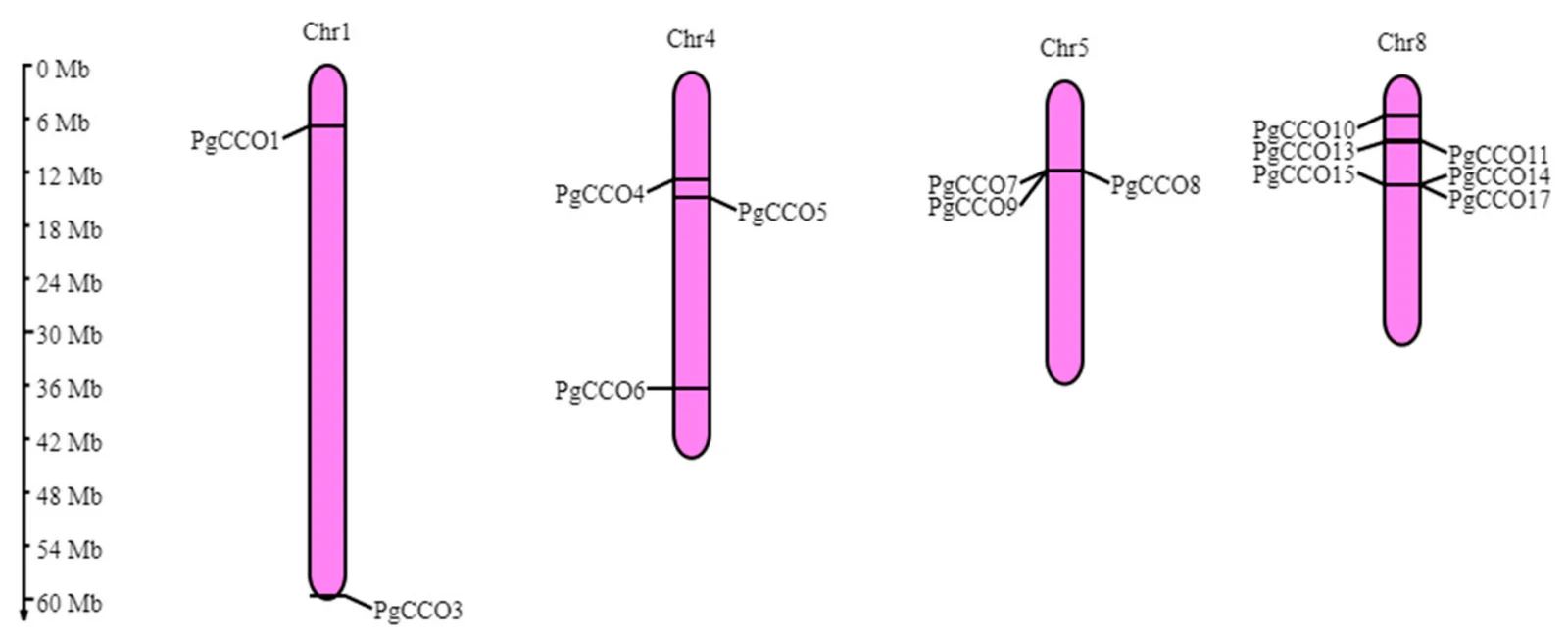
Chromosomal distribution of *PgCCO* genes in *P. granatum.* The vertical axis indicates chromosome length in megabases (Mb).

*P. granatum* also has eight chromosomes, similar to *P. dulcis*. *CCO* genes were identified only on chromosomes 1, 4, 5, and 8, with the highest number of *CCO* genes observed on chromosome 8. Three *CCO* genes were detected on the other chromosomes (Figure 5). The start positions of *PgCCO2* and *PgCCO3* on chromosome 1, and *PgCCO12* and *PgCCO13*, as well as *PgCCO16* and *PgCCO17* on chromosome 8, overlap, and therefore they are not shown in the figure (Supplementary Table S1).

### 3.3. Phylogenetic Analysis

**Figure 6:**
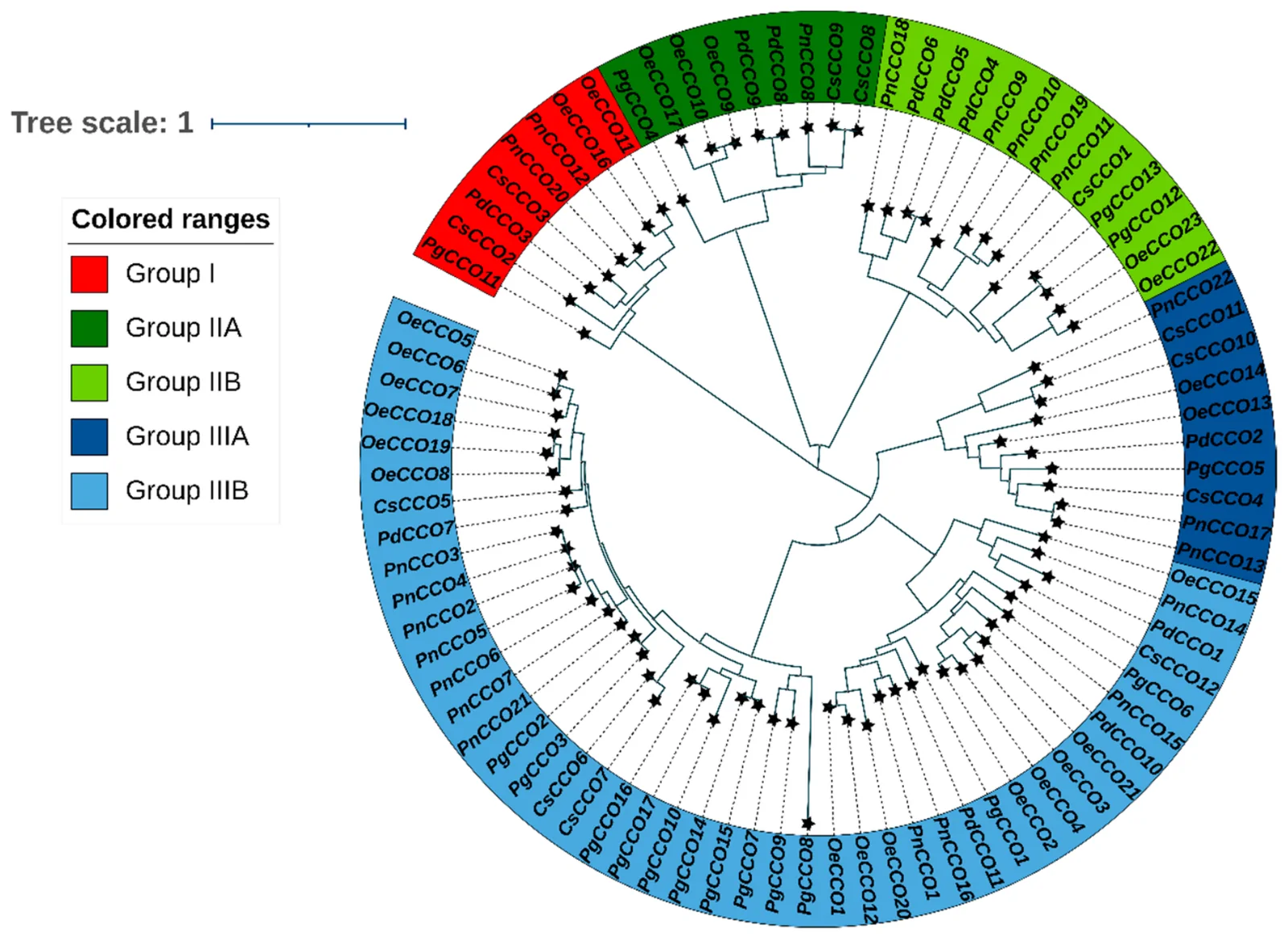
Phylogenetic relationships among CCO proteins from *C. sinensis*, *O. europaea* var. *sylvestris*, *P. nigra*, *P. dulcis*, and *P. granatum.* Black stars indicate supported internal nodes in the phylogenetic tree.

A phylogenetic tree was constructed for the *C. sinensis*, *O. europaea* var. *sylvestris*, *P. nigra*, *P. dulcis*, and *P. granatum* based on the results of the phylogenetic analysis. The phylogenetic tree of these species revealed three main groups, with Group II and Group III further divided into two sub-branches. The group with the highest number of members was Group III, while Group I had the fewest members (Figure 6).

### 3.4. Gene Structure and Conserved Protein Motif Analysis

**Figure 7:**
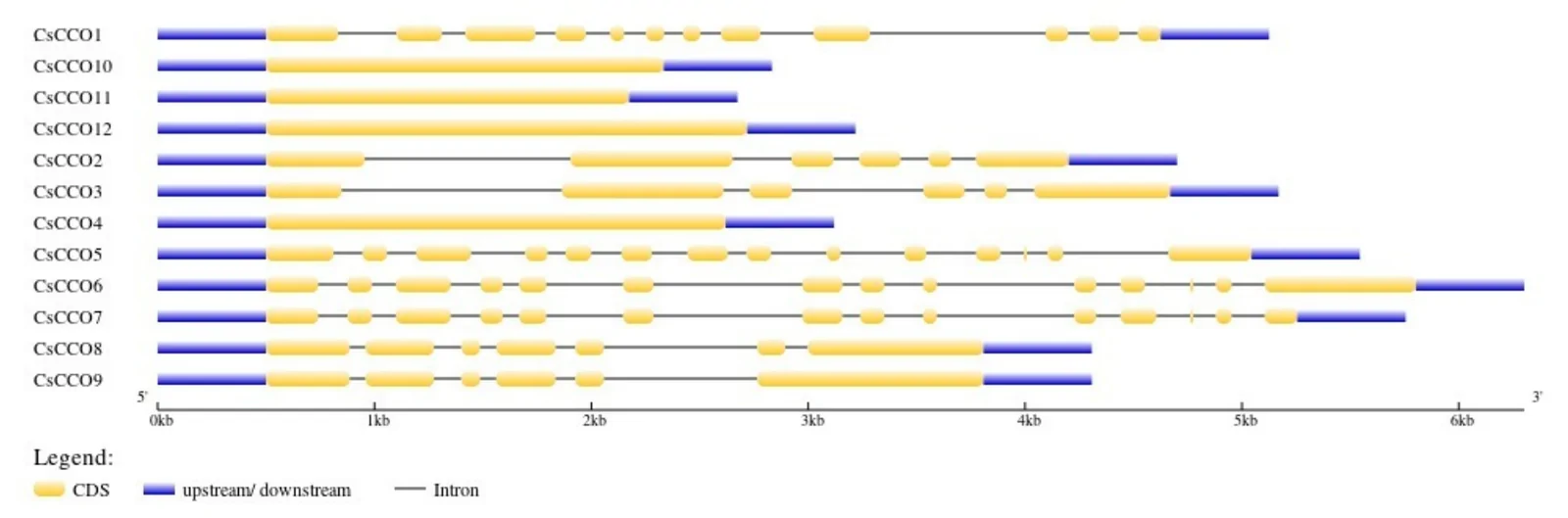
Gene structure of *CsCCO* genes.

As a result of the gene structure analysis, *CsCCO4*, *CsCCO10*, *CsCCO11*, and *CsCCO12* were found to lack introns. The longest gene was identified as *CsCCO16*, while the shortest was *CsCCO11*. Additionally, the genes with the highest number of introns and exons were *CsCCO5*, *CsCCO6*, and *CsCCO7* contained the highest number of exons (14) and introns (13). The longest exon was observed in *CsCCO12*. Both 5′-UTR and 3′-UTR regions were identified in all *CsCCO* genes (Figure 7).

**Figure 8:**
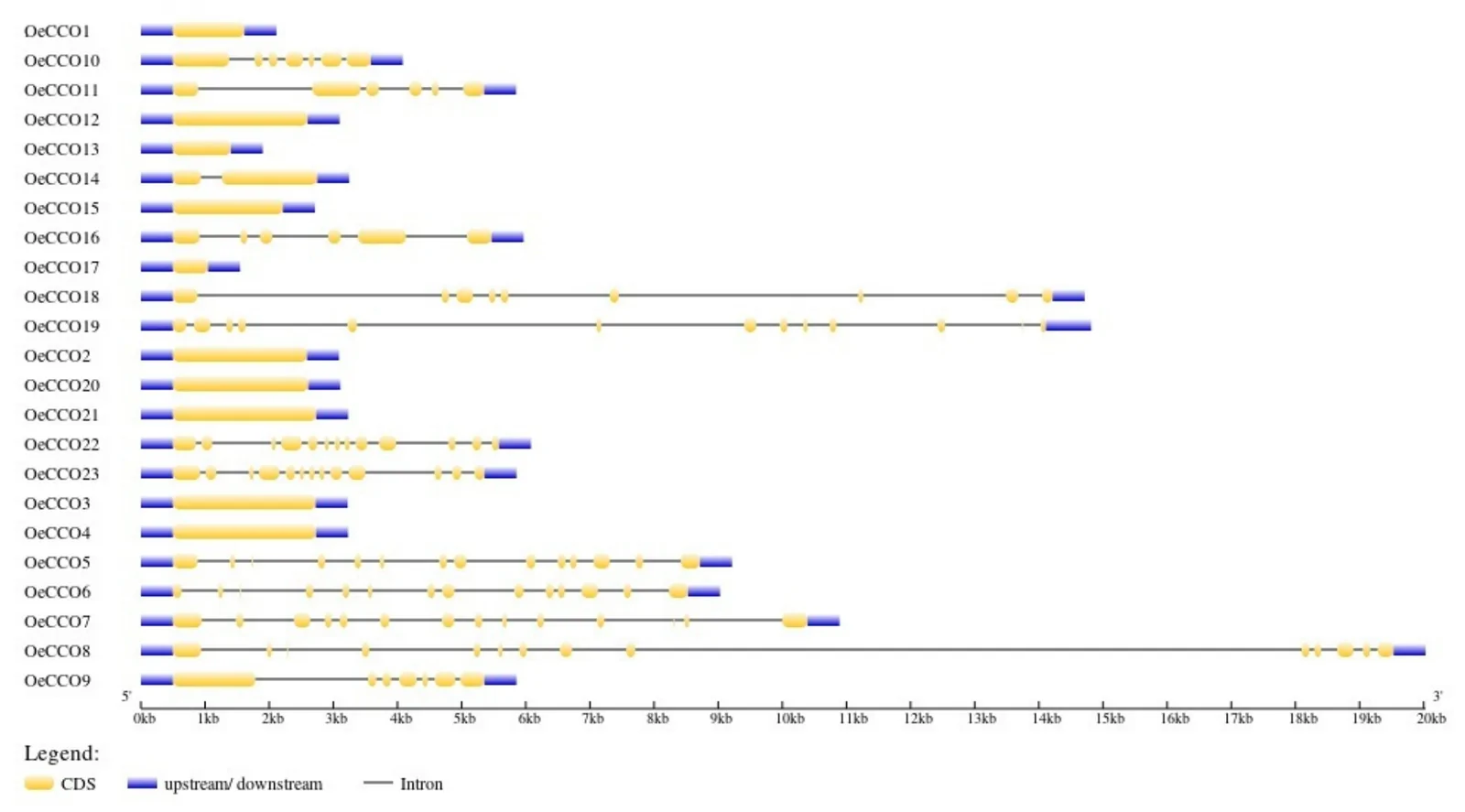
Gene structure of *OeCCO* genes.

In the gene structure analysis conducted on *O. europaea* var. *sylvestris*, no introns were detected in *OeCCO1*, *OeCCO2*, *OeCCO3*, *OeCCO4*, *OeCCO12*, *OeCCO13*, *OeCCO15*, *OeCCO17*, *OeCCO20*, and *OeCCO21*. The longest gene was identified as *OeCCO8*, while the shortest was *OeCCO17*. Notably, *OeCCO8* contained a particularly long intron. *OeCCO5*, *OeCCO6*, *OeCCO7*, and *OeCCO8* exhibited the highest exon and intron numbers, each containing 14 exons and 13 introns. Both 5′-UTR and 3′-UTR regions were identified in all *OeCCO* genes (Figure 8).

**Figure 9:**
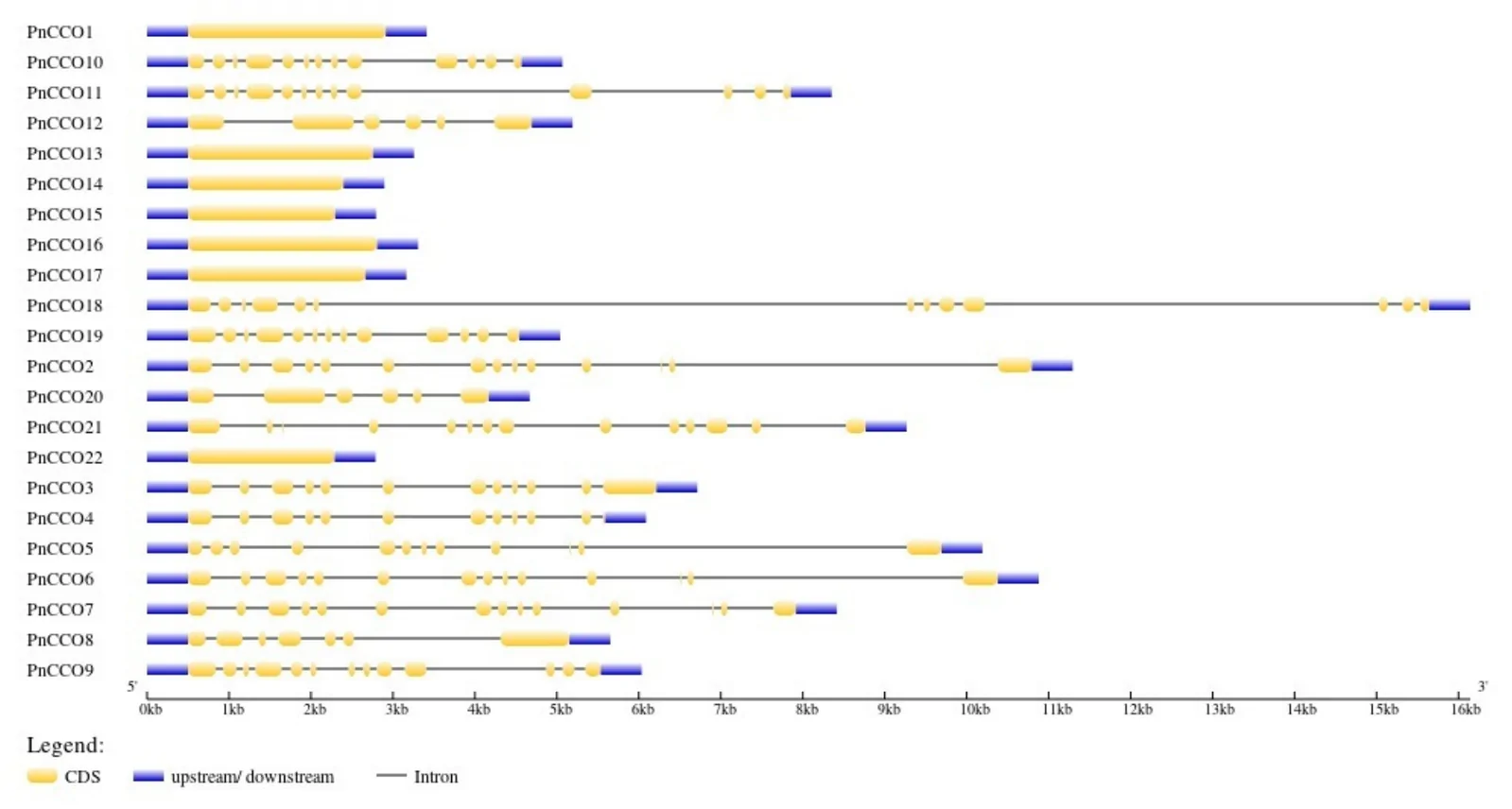
Gene structure of *PnCCO* genes.

In the gene structure analysis conducted on *P. nigra*, no introns were detected in the genes *PnCCO1*, *PnCCO13*, *PnCCO14*, *PnCCO15*, *PnCCO16*, *PnCCO17*, and *PnCCO22*. *PnCCO18* was observed to have the largest size, while *PnCCO15* and *PnCCO22* were comparatively smaller. Additionally, *PnCCO18* was found to contain two very long introns. The genes with the highest number of exons and introns were identified as *PnCCO2*, *PnCCO6*, *PnCCO7*, and *PnCCO21*. Moreover, 5′-UTR and 3′-UTR regions were observed in all genes (Figure 9).

**Figure 10:**
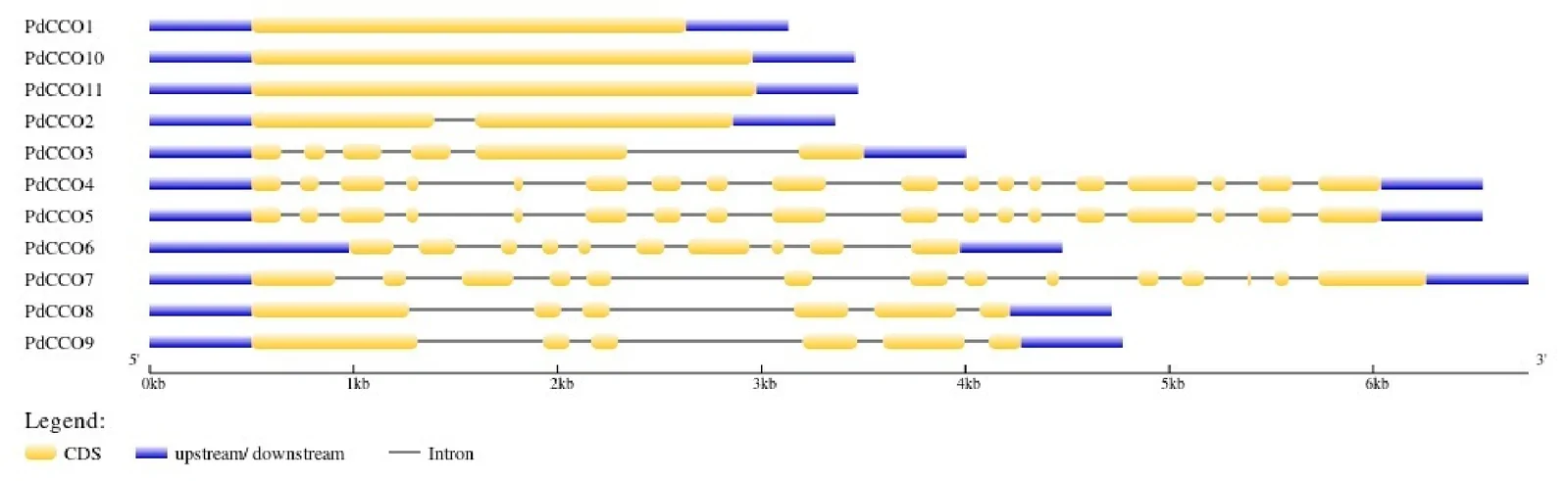
Gene structure of *PdCCO* genes.

According to the analysis results for *P. dulcis*, no introns were detected in *PdCCO1*, *PdCCO10*, and *PdCCO11*. *PdCCO7* was identified as the longest gene, while *PdCCO1* was observed to be the shortest. The genes with the highest number of exons and introns were identified as *PdCCO4* and *PdCCO5*. Moreover, 5′-UTR and 3′-UTR regions were observed in all genes (Figure 10).

**Figure 11:**
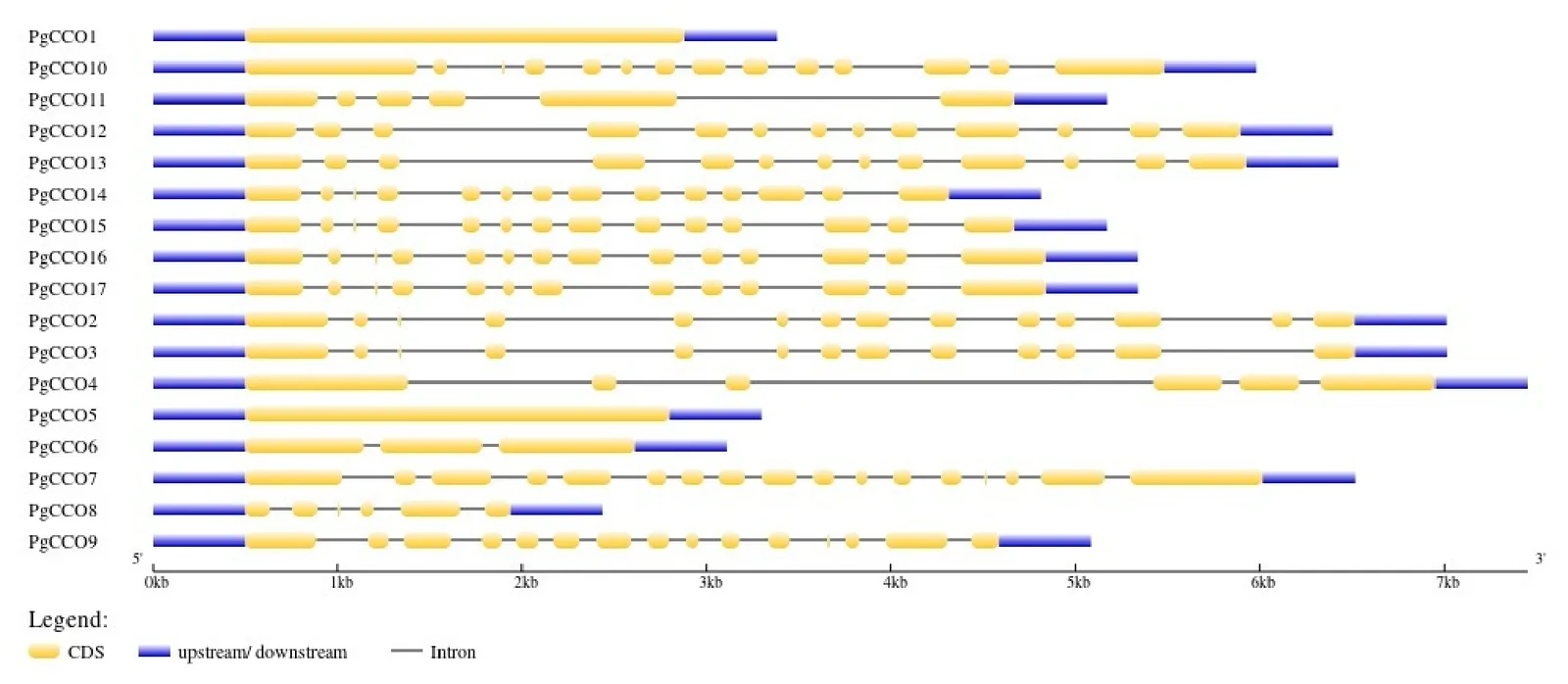
Gene structure of *PgCCO* genes.

In the gene structure analysis of *P. granatum*, no introns were detected in *PgCCO1* and *PgCCO5*. *PgCCO4* contained a remarkably long intron and was identified as the longest gene, whereas *PgCCO8* was the shortest. *PgCCO7* exhibited the highest number of exons and introns. Both 5′-UTR and 3′-UTR regions were identified in all *PgCCO* genes (Figure 11).

**Figure 12:**
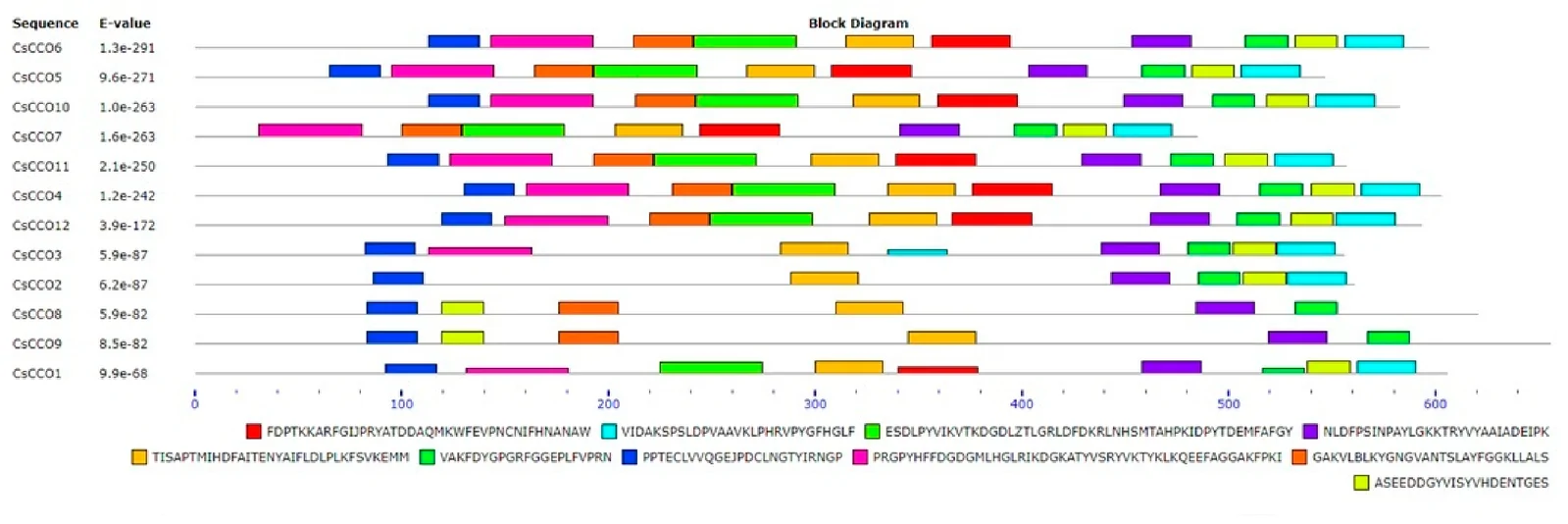
Distribution of conserved motifs in the CsCCO proteins. Different colored boxes indicate distinct conserved motifs.

Conserved motif analysis revealed that CsCCO4, CsCCO5, CsCCO6, CsCCO10, CsCCO11, and CsCCO12 contained all ten conserved motifs. Motif 9 was absent in CsCCO1, whereas motif 7 was missing in CsCCO7. In addition, motif 2 was duplicated in CsCCO3 (Figure 12).

**Figure 13:**
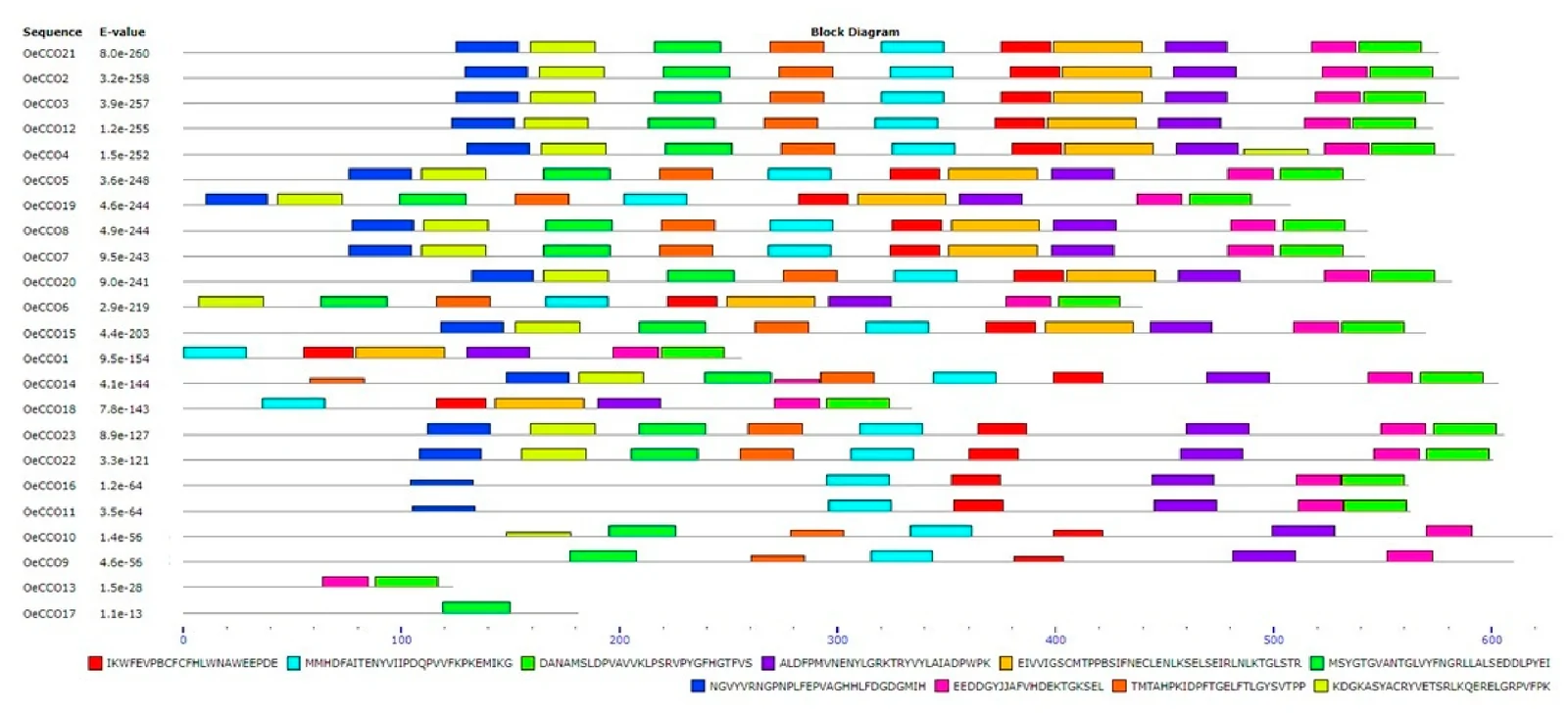
Distribution of conserved motifs in OeCCO proteins.

The conserved motif analysis of OeCCO proteins revealed that all ten conserved motifs were present in OeCCO2, OeCCO3, OeCCO4, OeCCO5, OeCCO7, OeCCO8, OeCCO12, OeCCO14, OeCCO15, OeCCO19, OeCCO20, and OeCCO21. Among these, OeCCO4 contained two copies of motif 10, whereas OeCCO14 contained two copies of motif 9. OeCCO6 lacked motif 7 and therefore contained nine motifs. Likewise, OeCCO22 and OeCCO23 each contained nine motifs due to the absence of motif 5. In contrast, OeCCO17 contained only motif 6 (Figure 13).

**Figure 14:**
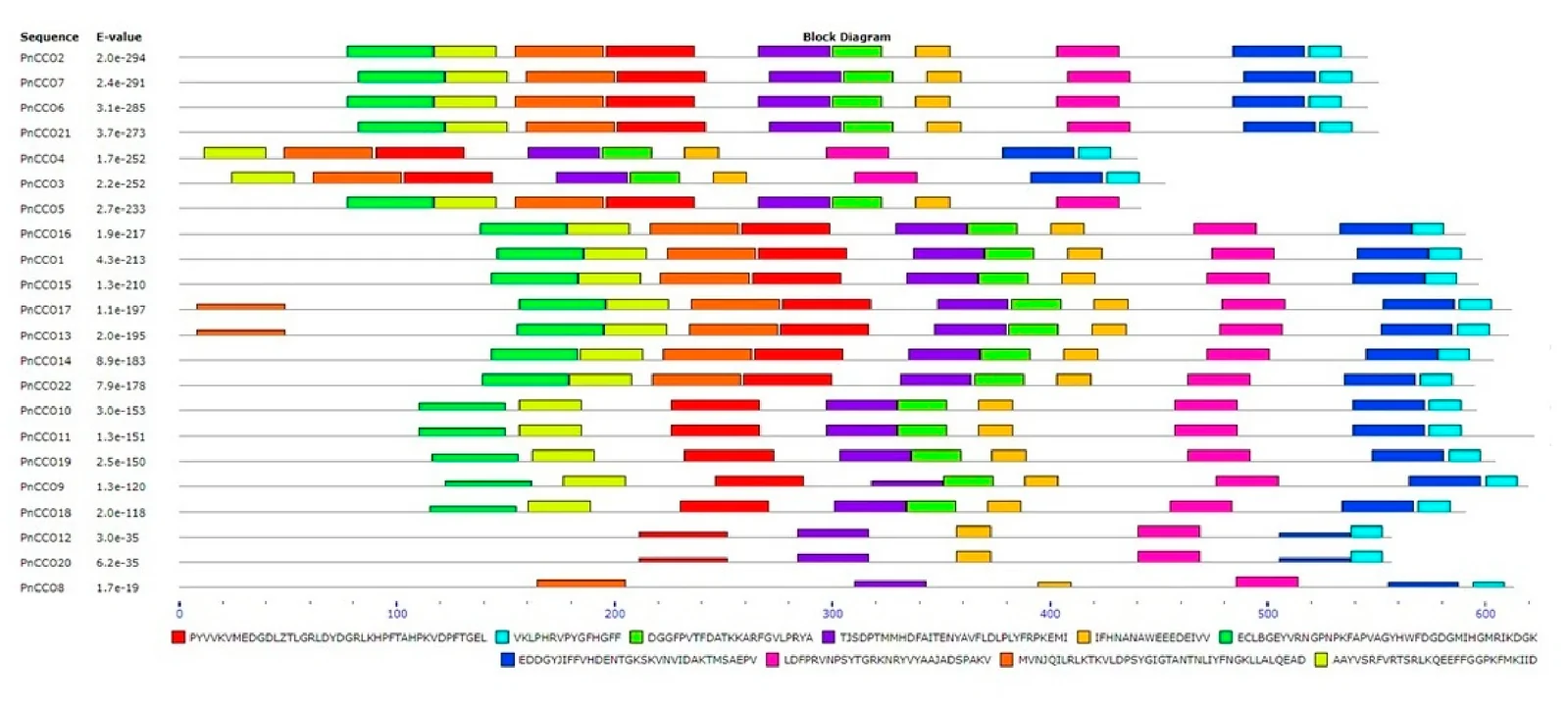
Distribution of conserved motifs in PnCCO proteins.

The conserved motif analysis of PnCCO proteins revealed that all ten conserved motifs were present in PnCCO1, PnCCO2, PnCCO6, PnCCO7, PnCCO13, PnCCO14, PnCCO15, PnCCO16, PnCCO17, PnCCO21, and PnCCO22. Among these, PnCCO13 and PnCCO17 each contained two copies of motif 9. In contrast, PnCCO3 and PnCCO4 lacked motif 6, whereas PnCCO9, PnCCO10, PnCCO11, PnCCO18, and PnCCO19 lacked motif 9, resulting in each of these proteins containing nine motifs (Figure 14).

**Figure 15:**
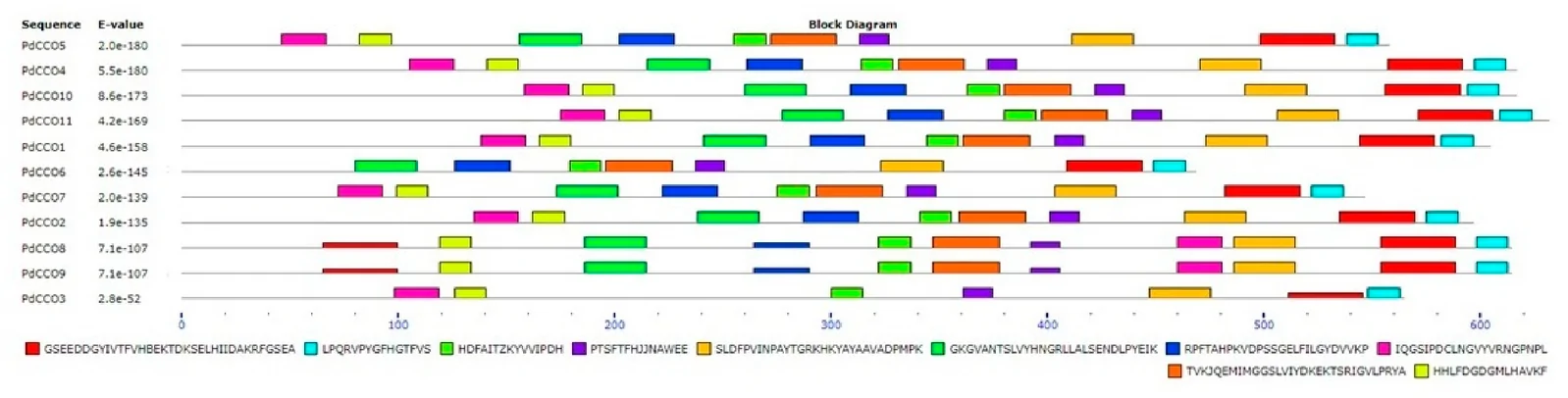
Distribution of conserved motifs in PdCCO proteins.

The conserved motif analysis of PdCCO proteins revealed that all ten conserved motifs were present in all proteins except PdCCO3 and PdCCO6. Additionally, PdCCO8 and PdCCO9 each contained two copies of motif 1. PdCCO3 lacked motifs 6, 7, and 9, whereas PdCCO6 lacked motifs 8 and 10 (Figure 15).

**Figure 16:**
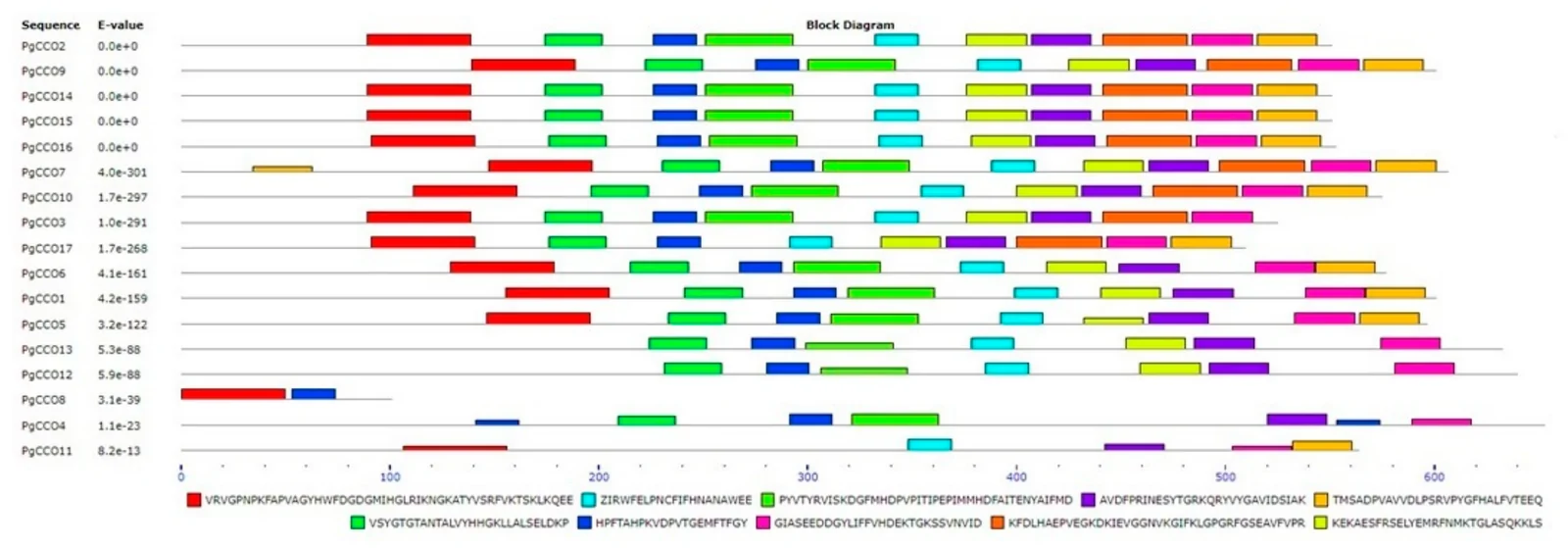
Distribution of conserved motifs in PgCCO proteins.

The conserved motif analysis of PgCCO proteins revealed that all ten conserved motifs were present in PgCCO2, PgCCO7, PgCCO9, PgCCO10, PgCCO14, PgCCO15, and PgCCO16. Among these, PgCCO7 contained two copies of motif 5. In contrast, PgCCO8 contained only motifs 1 and 7 (Figure 16).

### 3.5. Homology Modeling of CCO Proteins

**Figure 17:**
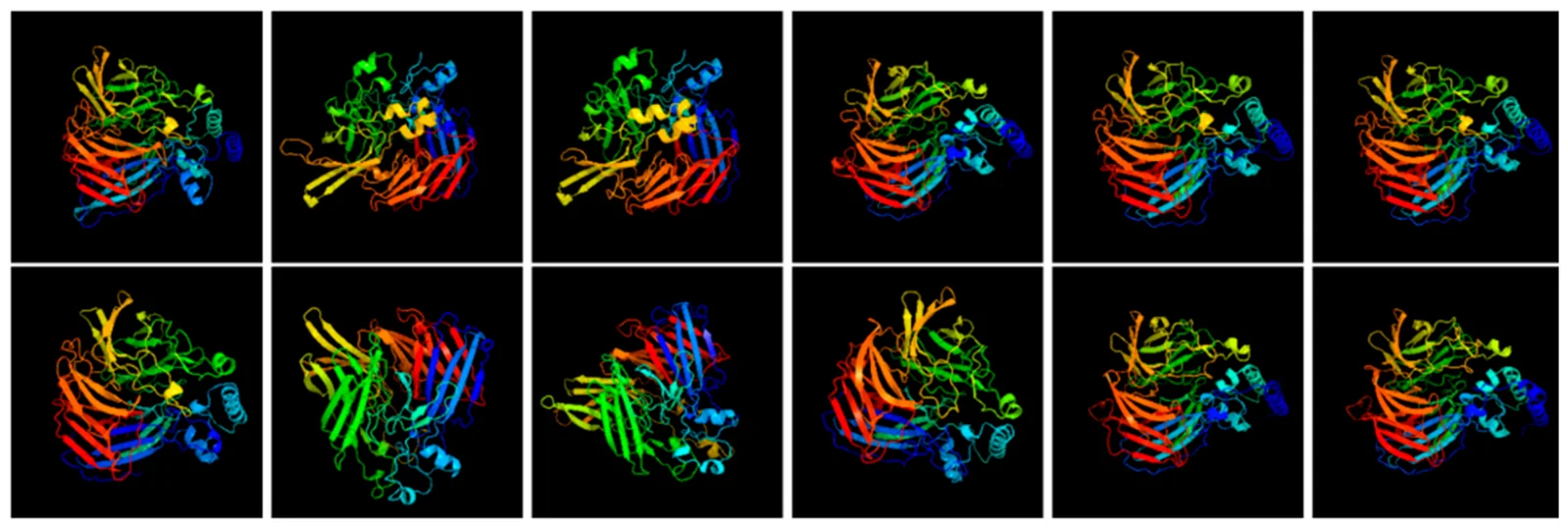
Three-dimensional structural models of CsCCO proteins arranged sequentially according to gene number (CsCCO1–CsCCO12). The models illustrate the overall structural organization of the proteins, including α-helices, β-sheets, and loop regions, highlighting the structural similarities and differences among the CsCCO proteins. The ribbon colors represent the protein sequence from the N-terminus (red) to the C-terminus (blue).

The intensive mode of the Phyre2 database was utilized for homology modeling, with the confidence level set to 90%. All CsCCO proteins were determined to have a confidence percentage of 100%. The analysis revealed that β sheets were predominant. Additionally, helices (α or transmembrane helices), antiparallel β sheets, β turns, and long loops were identified in all CsCCO proteins (Figure 17). Comprehensive analysis showed that α helices were absent in CsCCO8 and CsCCO9, while transmembrane helices were found at a rate of 3% in CsCCO8 and 2% in CsCCO9. Furthermore, the highest percentage of β strands (42%) was observed in CsCCO7.

**Figure 18:**
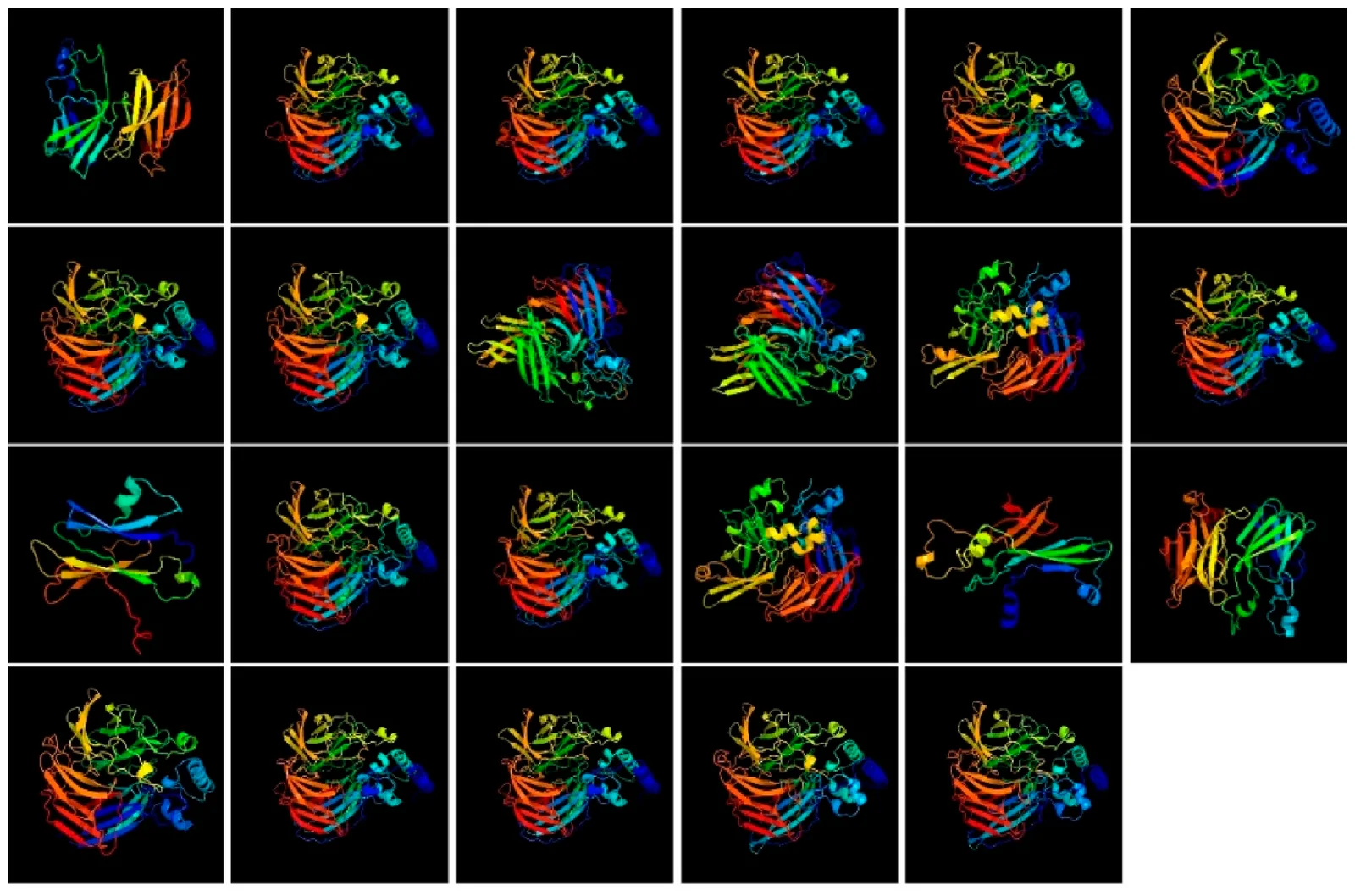
Three-dimensional structural models of OeCCO proteins arranged sequentially according to gene number (OeCCO1–OeCCO23). The models illustrate the overall structural organization of the proteins, including α-helices, β-sheets, and loop regions, highlighting the structural similarities and differences among the OeCCO proteins.ee-dimensional structures of OeCCO proteins arranged according to gene number. The ribbon colors represent the protein sequence from the N-terminus (red) to the C-terminus (blue).

In the analysis of OeCCO proteins, all proteins were observed to have a confidence percentage of 100%, with β-sheets being the most prevalent structural feature. Most OeCCO proteins were found to possess α-helices, antiparallel β-sheets, β-turns, and long loops (Figure 18). The highest proportion of β-strands (47%) was identified in OeCCO6 and OeCCO13, followed by OeCCO18 (46%) and OeCCO1 (45%). Transmembrane helices were detected in all OeCCO proteins except OeCCO1, OeCCO11, OeCCO13, OeCCO16, OeCCO17, and OeCCO18. Among all OeCCO proteins, OeCCO7 exhibited the highest percentage of α-helices, calculated at 8%.

**Figure 19:**
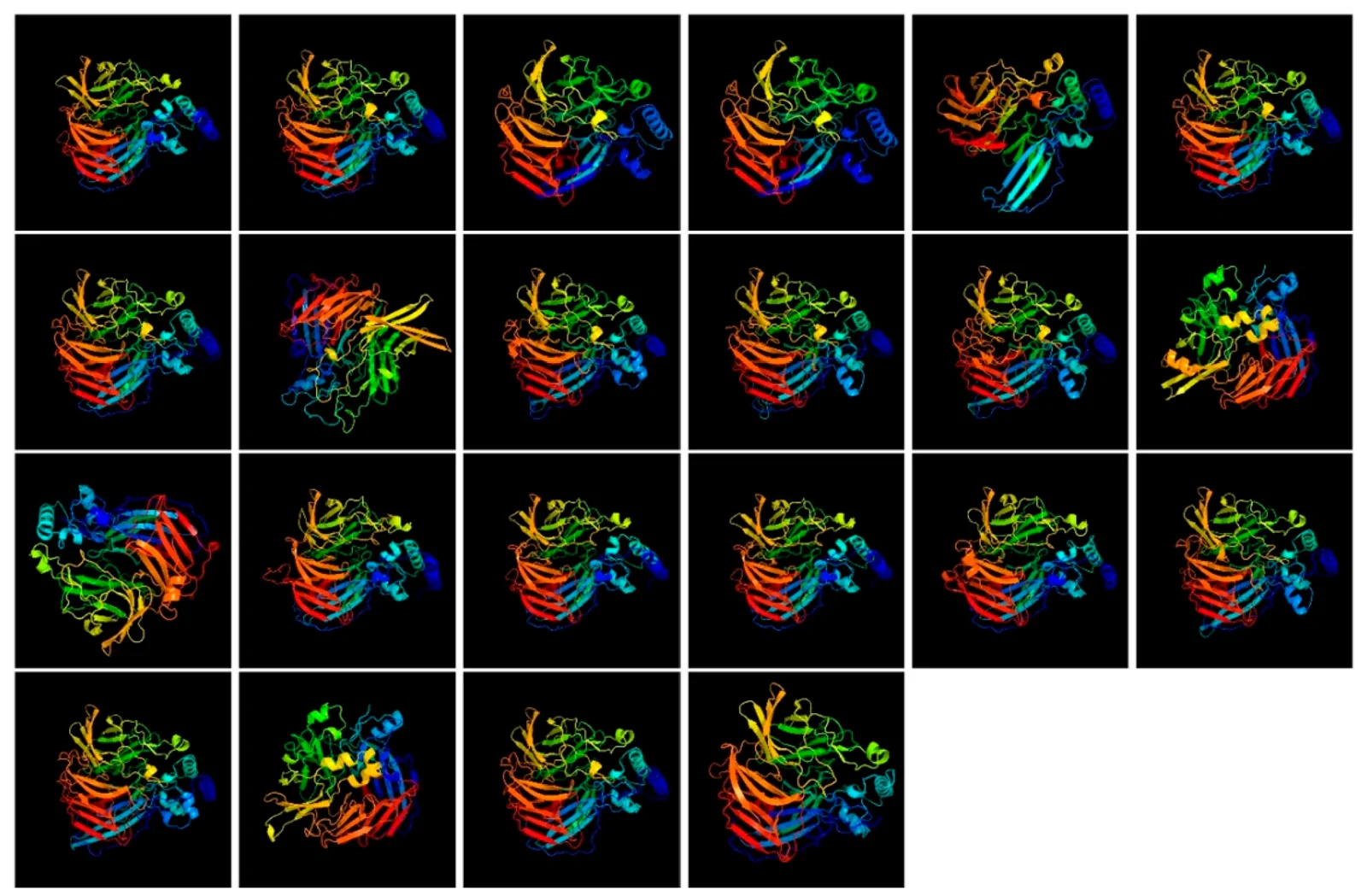
Three-dimensional structural models of PnCCO proteins arranged sequentially according to gene number (PnCCO1–PnCCO22). The models illustrate the overall structural organization of the proteins, including α-helices, β-sheets, and loop regions, highlighting the structural similarities and differences among the PnCCO proteins. The ribbon colors represent the protein sequence from the N-terminus (red) to the C-terminus (blue).

The analysis revealed that the confidence percentage was 100% for all proteins. In PnCCO proteins, β-sheets were prominently observed. Except for PnCCO8, α-helices were identified in all proteins, while other structures (antiparallel β-sheets, β-turns, and long loops) were detected in all proteins (Figure 19). Although α-helices were not identified in PnCCO8, transmembrane helices were observed. Conversely, PnCCO20 lacked transmembrane helices. Additionally, the highest percentage of β-strands was found in PnCCO3 (46%), followed by PnCCO3 at 45%.

**Figure 20:**
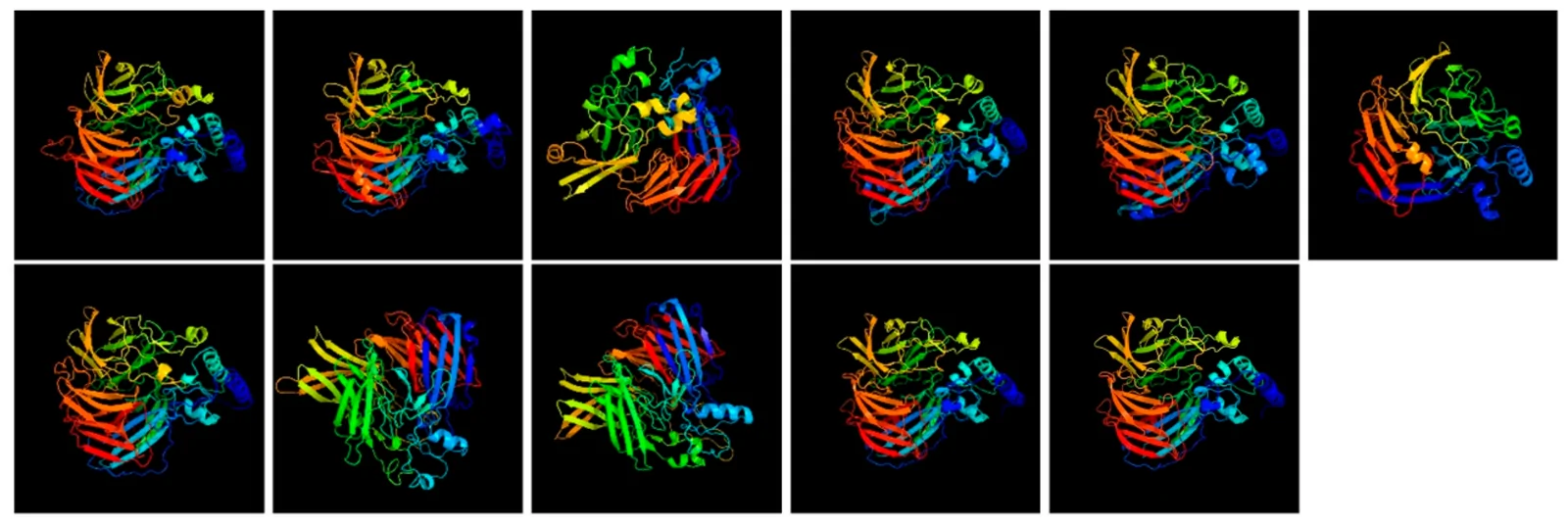
Three-dimensional structural models of PdCCO proteins arranged sequentially according to gene number (PdCCO1–PdCCO11). The models illustrate the overall structural organization of the proteins, including α-helices, β-sheets, and loop regions, highlighting the structural similarities and differences among the PdCCO proteins. The ribbon colors represent the protein sequence from the N-terminus (red) to the C-terminus (blue).

The analysis revealed that the confidence percentage was determined to be 100% for all PdCCO proteins. Helices (α or transmembrane helices), antiparallel β sheets, β turns, and long loops were observed in all proteins, with antiparallel β sheets being particularly predominant (Figure 20). Additionally, it was determined that transmembrane helices are absent in PdCCO3. Moreover, β-strands were found to be most abundant (41%) in PdCCO3 and PdCCO6, while the highest percentage of α helices (7%) was observed in PdCCO7.

**Figure 21:**
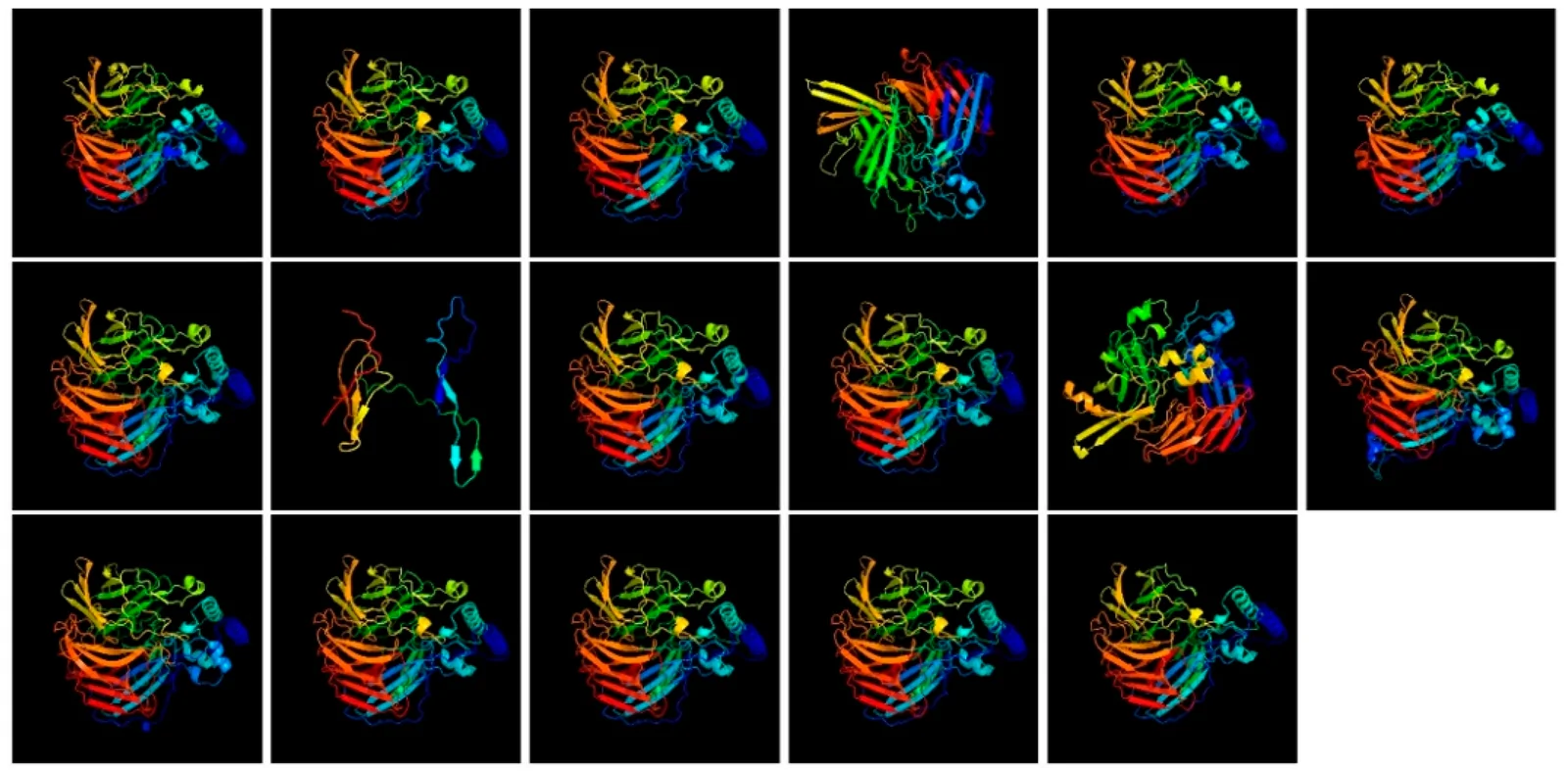
Three-dimensional structural models of PgCCO proteins arranged sequentially according to gene number (PgCCO1–PgCCO17). The models illustrate the overall structural organization of the proteins, including α-helices, β-sheets, and loop regions, highlighting the structural similarities and differences among the PgCCO proteins. The ribbon colors represent the protein sequence from the N-terminus (red) to the C-terminus (blue).

The confidence percentages for all PgCCO proteins were found to be 100%, as observed in other species. β sheets were predominant in PgCCOs, and α-helices, antiparallel β-sheets, β-turns, and long loops were identified in all PgCCO proteins except PgCCO8. In PgCCO8, neither α-helices nor transmembrane helices were observed (Figure 21). Additionally, transmembrane helices were not detected in PgCCO11. Furthermore, the highest proportion of β-strands (42%) was found in PgCCO11, while the lowest was identified in PgCCO4 (29%). Moreover, the highest percentage of α-helices (10%) was observed in PgCCO10.

### 3.6. Protein Subcellular Localization Analysis

**Figure 22:**
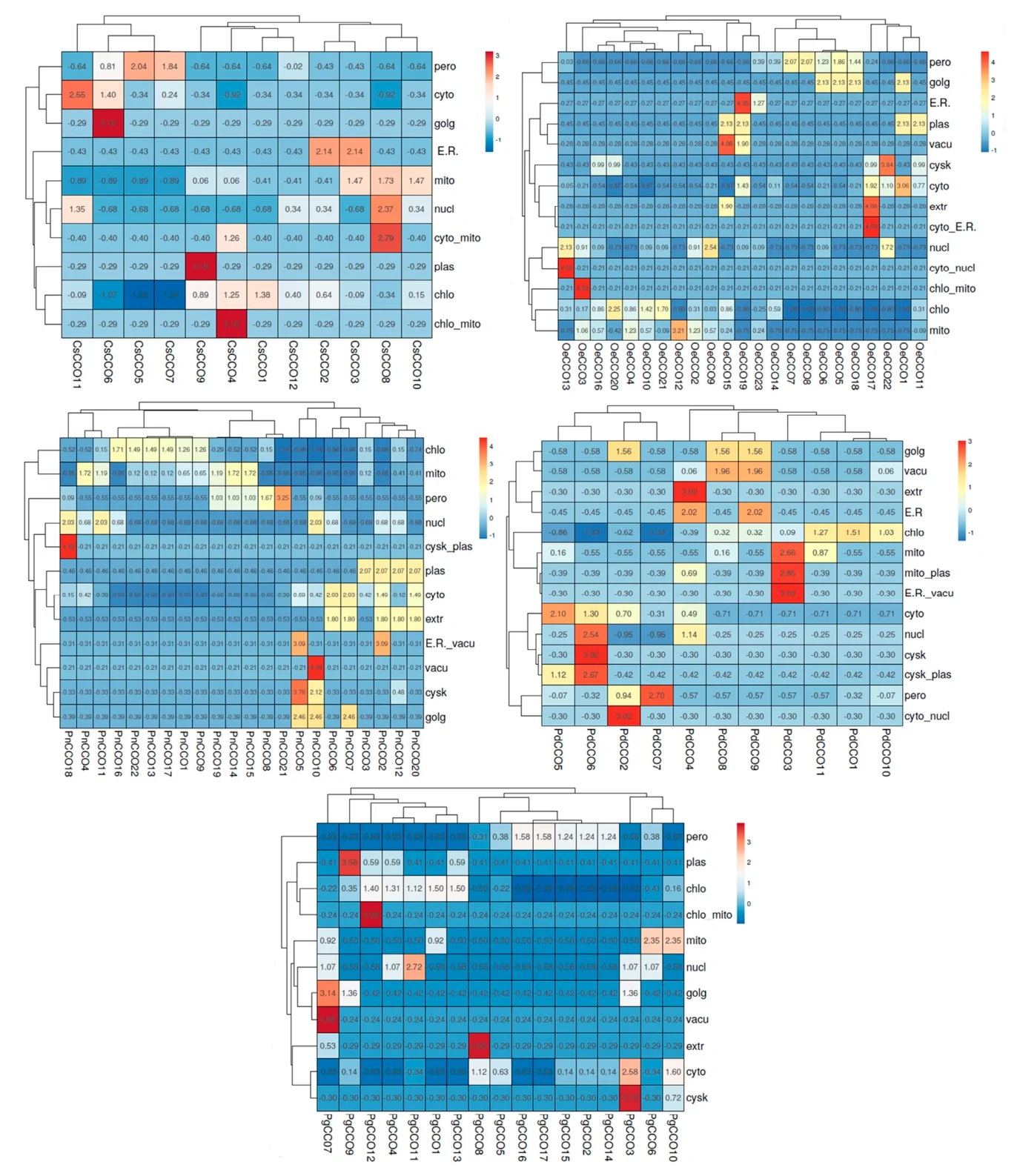
Predicted subcellular localization of CCO proteins in *C. sinensis*, *O. europaea* var. *sylvestris*, *P. nigra*, *P. dulcis*, and *P. granatum*. The heat map illustrates the predicted localization patterns of CCO proteins across different cellular compartments based on computational analysis. Color intensity represents the relative prediction score for localization in each compartment. Abbreviations: chlo, chloroplast; mito, mitochondrion; pero, peroxisome; cyto, cytoplasm; nucl, nucleus; golg, Golgi apparatus; plas, plasma membrane; ER, endoplasmic reticulum; vacu, vacuole; extr, extracellular space; cysk, cytoskeleton. Dual-localization compartments include chlo_mito, cyto_mito, cyto_nucl, cyto_ER, ER_vacu, cysk_plas, and mito_plas.

The predicted subcellular localization analysis revealed that CCO proteins were distributed across diverse cellular compartments, including the chloroplast, mitochondrion, peroxisome, cytoplasm, nucleus, Golgi apparatus, plasma membrane, endoplasmic reticulum, vacuole, extracellular space, cytoskeleton, and several dual-localization compartments (Figure 22). Overall, chloroplasts and mitochondria were the predominant predicted localization sites across all five species, whereas localization to the cytoskeleton, vacuole, extracellular space, and dual-compartment locations was limited to a relatively small number of proteins.

Comparative analysis revealed both conserved and species-specific localization patterns. Members of the CsCCO, OeCCO, PnCCO, PdCCO, and PgCCO families were commonly predicted to localize to the chloroplast, mitochondrion, cytoplasm, nucleus, Golgi apparatus, and peroxisome. In contrast, plasma membrane localization was absent in PdCCO proteins, chloroplast–mitochondrion dual localization was not detected in PnCCO or PdCCO proteins, and endoplasmic reticulum localization was absent in PnCCO and PgCCO proteins. Furthermore, CsCCO proteins showed no predicted localization to the cytoskeleton, vacuole, or extracellular space, whereas cytoplasm–nucleus dual localization was not observed in the CsCCO, PnCCO, or PgCCO families. Endoplasmic reticulum–vacuole and cytoskeleton–plasma membrane dual localization occurred only in selected OeCCO, PnCCO, and PdCCO proteins.

Species-specific differences were also evident. CsCCO proteins exhibited relatively simple localization profiles, with only a few members predicted to localize to the Golgi apparatus, plasma membrane, endoplasmic reticulum, or chloroplast–mitochondrion dual compartment. Among the five species, OeCCO proteins displayed the greatest diversity of predicted localization patterns, including localization to the cytoskeleton, vacuole, extracellular space, cytoplasm–endoplasmic reticulum, and cytoplasm–nucleus dual compartments. PnCCO proteins uniquely exhibited endoplasmic reticulum–vacuole and cytoskeleton–plasma membrane dual localization, whereas PdCCO proteins were the only family with members predicted to localize to the mitochondrion–plasma membrane compartment. In contrast, PgCCO proteins were predominantly localized to the chloroplast, mitochondrion, nucleus, Golgi apparatus, plasma membrane, and extracellular space, with only a single protein predicted to exhibit chloroplast–mitochondrion dual localization.

### 3.7. Cis-Acting Elements Analysis

**Figure 23:**
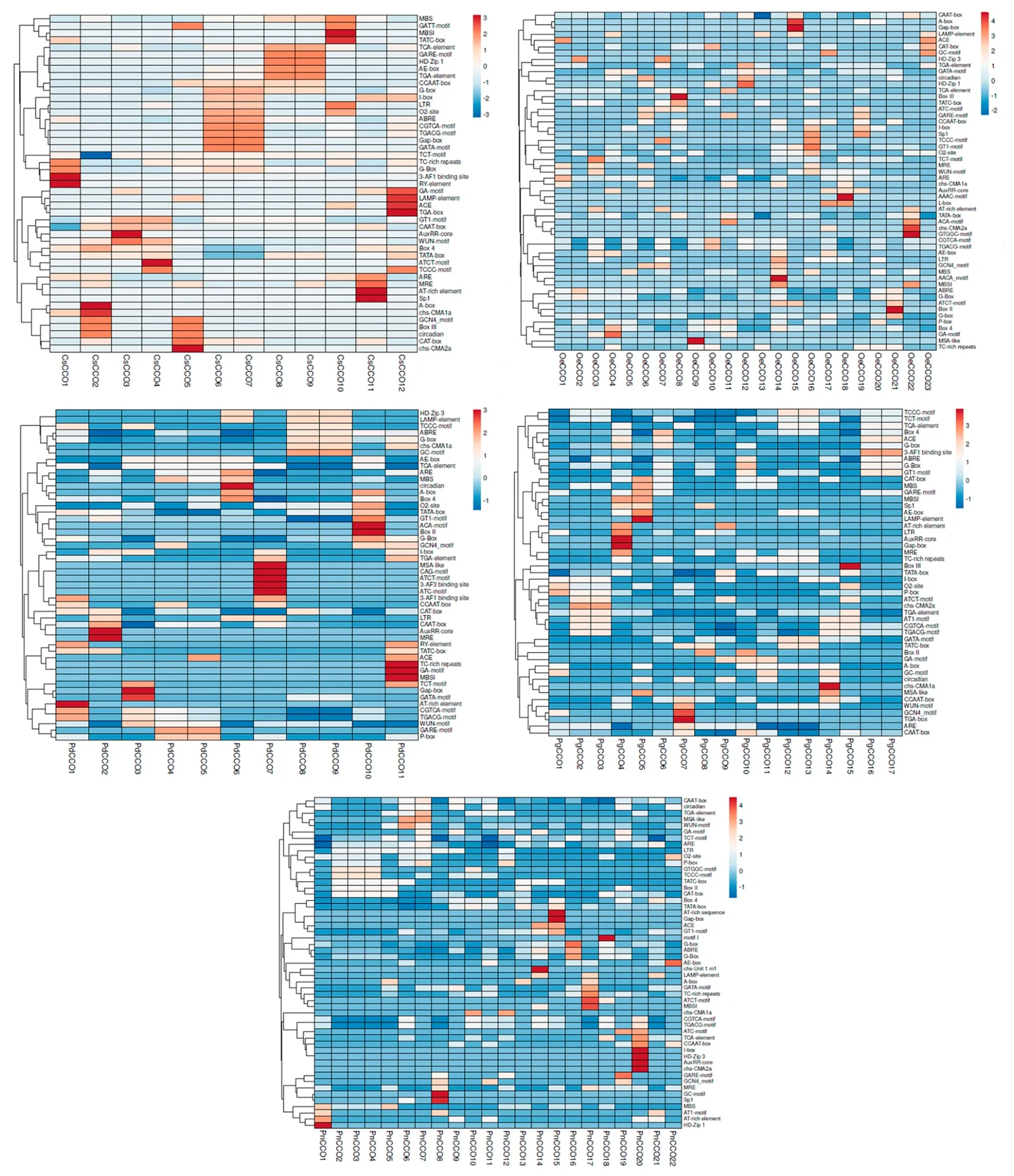
Heat maps showing the distribution of cis-acting regulatory elements in the promoter regions of *CCO* genes.

As a result of the cis-acting element analysis performed on the promoter regions of the *CsCCO*, *OeCCO*, *PnCCO*, *PdCCO*, and *PgCCO* genes, the identified motifs with known names and functions were tabulated, and their numbers were calculated (Supplementary Table S2). Based on these counts, a heat map was generated (Figure 23). Based on the data obtained, *CsCCO12* (144) contained the highest number of cis-acting elements among the *CsCCO* genes, followed by *CsCCO2* (139) and *CsCCO4* (138) in second and third place, respectively. In addition, it was determined that the highest number of cis-acting elements in the *OeCCO* genes was found in *OeCCO22* (153), followed by *OeCCO15* (131) and *OeCCO11* (129). Additionally, among the *PnCCO* genes, the gene with the highest number of cis-acting elements was observed to be *PnCCO15* (171), followed by *PnCCO13* (161) and *PnCCO10* (157). Furthermore, among the *PdCCO* genes, *PdCCO1* (165) was found to have the highest number of cis-acting elements, followed by *PdCCO10* (162) and *PdCCO2* (149). Finally, in the *PgCCO* genes, *PgCCO15* (161) had the highest number of cis-acting elements, followed by *PgCCO4* (158) and *PgCCO11* (149).

When examined in terms of motifs, 47 different motifs were identified in *CsCCO*, 54 in *OeCCO*, 53 in *PnCCO*, 50 in *PdCCO*, and 50 in *PgCCO*. The *CsCCO*, *OeCCO*, *PnCCO*, *PdCCO*, and *PgCCO* genes were found to contain motifs such as LTR (low temperature), MBS (drought), TC-rich repeats (defense and stress), and WUN-motifs (wound), which are associated with stress responses. In addition, it was determined that these genes predominantly contain motifs related to light response compared to other motifs. These motifs are as follows: 3-AF1 binding site, AAAC-motif, ACA-motif, ACE, AE-box, AT1-motif, ATC-motif, ATCT-motif, Box II, Box 4, CAG-motif, chs-CMA1a, chs-CMA2a, chs-Unit 1 m1, G-box, G-Box, GA-motif, Gap-box, GC-motif, GATA-motif, GATT-motif, GT1-motif, GTGGC-motif, I-box, LAMP-element, MRE, Sp1, TCCC-motif, TCT-motif (Supplementary Table S2).

Phytohormones play key roles in stress mechanisms, and through their interactions, these hormones regulate gene expression and ensure the formation of stress responses [43]. Motifs related to phytohormones such as ABRE (abscisic acid), AuxRR-core (auxin), CGTCA-motif (methyl jasmonate), GARE-motif (gibberellin), TATC-box (gibberellin), TCA-element (salicylic acid), TGA-element (auxin), and TGACG-motif (methyl jasmonate) have been identified in the *CsCCO*, *OeCCO*, *PnCCO*, *PdCCO*, and *PgCCO* genes. Additionally, the TGA-box (auxin) motif has been determined only in the *CsCCO* and *PgCCO* genes, while the P-box (gibberellin) and GC-motif (gibberellin) motifs have been observed in all genes except *CsCCO* (Supplementary Table S2).

It was also determined that the *CsCCO*, *OeCCO*, *PnCCO*, *PdCCO*, and *PgCCO* genes contain motifs associated with important functions such as anaerobic induction, meristem expression, circadian control, endosperm expression, differentiation of the palisade mesophyll cells, regulation of flavonoid biosynthetic genes, seed-specific regulation, and cell cycle regulation (Supplementary Table S2).

### 3.8. miRNA Analysis

The complete miRNA target prediction results for the five species are presented in Supplementary Table S3. Considerable variation was observed in the number of predicted miRNA interactions among *CCO* genes across the five species. In *Citrus sinensis*, *CsCCO10* exhibited the highest number of predicted miRNA interactions (138), whereas *CsCCO8* showed the fewest (80). Similarly, *OeCCO16* (315), *PnCCO1* (379), *PdCCO2* (450), and *PgCCO12/PgCCO13* (238 each) were identified as the most frequently predicted targets within their respective species. In contrast, *OeCCO17* (9), *PnCCO15* (66), *PdCCO3* (54), and *PgCCO6/PgCCO9* (77 each) exhibited the lowest numbers of predicted miRNA interactions. These findings indicate substantial variation in the predicted post-transcriptional regulation of *CCO* genes across the five species, with some genes appearing to be subject to more extensive miRNA-mediated regulation than others.

Several conserved plant miRNA families, including miR156, miR159, miR160, miR162, miR165, miR166, miR167, miR168, miR169, miR170, miR171, miR172, miR319, miR390, miR393, miR395, miR396, miR397, miR398, miR408, miR474, miR528, miR529, miR845, miR851, miR854, miR896, miR901, miR903, miR1030, miR1035, miR1050, miR1088, miR1125, and miR1126 have previously been reported to participate in plant stress responses [44,45,46,47,48,49,50]. Among these, miR156, miR159, miR166, miR167, miR169, miR171, miR393, and miR395 were predicted to target multiple members of the *CCO* gene family across all five species, indicating broad target distributions. In contrast, miR170, miR845, miR851, miR854, and miR1088 were predicted to target only one or a few *CCO* genes, whereas no putative targets were identified for miR896, miR901, miR903, miR1030, miR1035, miR1050, miR1125, or miR1126 in any of the analyzed species. Overall, these results suggest that members of the *CCO* gene family may be differentially regulated by both conserved and species-specific miRNA families across the five species.

## 4. Discussion

As a result of the analyses conducted in *Citrus sinensis*, *Olea europaea* var. *sylvestris*, *Populus nigra*, *Prunus dulcis*, and *Punica granatum*, 12, 23, 22, 11, and 17 *CCO* genes were identified, respectively. Among the analyzed species, *O. europaea* var. *sylvestris* had the highest number of identified *CCO* genes, while *P. dulcis* had the lowest. Similarly, *C. sinensis* was found to have a *CCO* gene count close to that of *P. dulcis*. The differences in the number of *CCO* genes among these species provide valuable insights into their mechanisms for coping with environmental stresses and adapting to changing conditions. In particular, the highest number of *CCO* genes identified in *O. europaea* var. *sylvestris* suggests that this species may exhibit a higher degree of genetic diversity and adaptation potential to environmental changes and stress factors. Furthermore, it can be associated with an adaptation strategy that allows this species to become more resilient to variable environmental conditions, such as drought and high-temperature stress, particularly in the Mediterranean region. The ability of this species to regulate *CCO* gene expression in response to environmental conditions could be a key factor in enhancing its resilience. In contrast, the lower number of *CCO* genes in *P. dulcis* might reflect a distinct biological strategy in response to environmental stimuli. These variations point to the possibility that *CCO* genes are involved in shaping the different biological responses of plant species to environmental stresses. Studies on *CCO* genes have reported the following numbers: 47 in *Saccharum spontaneum*, 14 in *Saccharum* spp. R570 cultivar, 21 in *Malus domestica*, 15 in *Gossypium arboreum* and *G. raimondii*, 30 in *G. hirsutum*, 16 in *Oryza sativa* Japonica Group, 9 in *Cerasus humilis* (accepted name: *Prunus humilis* Bunge), 21 in *Helianthus annuus*, 12 in *Pyrus bretschneideri*, 11 in *Fragaria vesca*, 8 in *Prunus mume*, 10 in *P. persica*, 21 in *Coffea arabica*, 24 in *Arachis hypogaea*, 16 in *Betula platyphylla*, 15 in *Litchi chinensis*, 10 in *Cucumis sativus*, 15 in *Citrus clementina* Hort., and 10 in *Liriodendron chinense* [2,3,4,6,9,11,12,13,14,15,17,18,51,52]. The presence of 15 *CCO* genes in *C. clementina* [51], one of the species analyzed in the mentioned studies, aligns with the identification of 12 *CCO* genes in *C. sinensis*, which belongs to the same genus in the current study. Similarly, the number of *CCO* genes detected in *Pyrus bretschneideri*, *Fragaria vesca*, *Prunus mume*, *P. persica*, and *Prunus humilis* [3,6,52], all members of the Rosaceae family, is comparable to the number found in *P. dulcis*, which also belongs to the same family in the present study. However, although *Malus domestica* [3] is also a member of the Rosaceae family, it contains a higher number of *CCO* genes than the aforementioned species. The similar number of *CCO* genes in *P. dulcis* compared to other members of the Rosaceae family suggests that species within this family share a common genetic framework. However, the higher number of *CCO* genes in *M. domestica* indicates that this species may have followed a distinct evolutionary path regarding stress tolerance and metabolic regulation. The elevated *CCO* gene count in *M. domestica* may reflect more complex stress response mechanisms, suggesting that this species possesses a stronger ability to adapt to environmental changes.

The isoelectric point (pI) values of the CCO proteins analyzed in this study were found to range between 4.81 and 9.01. The highest pI value was observed in PnCCO15, while the lowest was found in OeCCO13. Additionally, the pI value range of OeCCOs (4.81–8.95) was broader compared to other species, whereas PdCCOs exhibited the narrowest range (5.37–6.97). Another noteworthy observation is that all PdCCOs have an acidic character. The broader pI value range of OeCCO proteins compared to other species suggests that these proteins may exhibit higher functional compatibility across various cellular environments and that this species could possess greater genetic diversity and adaptation potential in response to environmental changes and stress factors. In contrast, the clustering of pI values within a narrow range and the acidic character of all CCO proteins in *P. dulcis* may indicate that this species could develop more acidic responses to environmental stress conditions. Considering all CCO proteins, although they are generally acidic, some were determined to be basic. This suggests that CCO proteins may be directed to different cellular regions and participate in various biochemical processes. It has been reported that [53] the pI values of proteins are associated with subcellular localization, protein length, and the ecological niche of the organism. A length difference of 171 aa was found among CsCCO proteins, 504 aa among OeCCO proteins, 185 aa among PnCCO proteins, 192 aa among PdCCO proteins, and 552 aa among PgCCO proteins. These results indicate a significant variation in the length of CCO proteins among species. The widest variation was observed in PgCCO proteins, while the narrowest variation was found in CsCCO proteins. Additionally, the protein with the shortest amino acid sequence was identified as PgCCO8, while the longest sequence was observed in CsCCO9. A similar distribution was also observed in terms of molecular weight; the protein with the lowest molecular weight was PgCCO8, while the highest molecular weight was associated with CsCCO9. In the *P. granatum*, the difference in molecular weights spans a wider range compared to other species, while this difference is observed within a narrower range in *P. dulcis*. In terms of molecular weight and amino acid count, similar results were expected; however, the molecular weight scale is the narrowest in *P. dulcis*, while the narrowest variation in amino acid count is found in *C. sinensis*. Overall, the results indicate that the widest variation in both molecular weight and amino acid count is observed in *P. granatum*. These differences may also be related to the evolutionary history of the *CCO* gene family in related species, genome sizes, and gene duplication events. The significant variation observed, particularly in species like *P. granatum*, suggests that the genetic diversity and adaptation processes in these species may be more dynamic. This high variation may reflect the ability of these species to evolve more flexible protein functions, enhancing their capacity to adapt to environmental stresses. Additionally, gene duplication events may have contributed to the diversification of the CCO protein family, allowing these species to develop more specialized or diverse functions that support their survival and reproduction under different ecological conditions.

These findings indicate that the functional diversity of CCO proteins is associated with genetic mechanisms that enhance genetic diversity and environmental adaptation. Specifically, the differences in the chromosomal distribution of *CCO* genes could provide deeper insights into the evolutionary adaptations of species and their strategies for responding to environmental stresses. In the current study, the distribution analysis of *CCO* genes revealed that these genes are concentrated on certain chromosomes. For example, in *C. sinensis*, they are found predominantly on chromosome 4; in *O. europaea* var. *sylvestris*, on chromosome 15; in *P. nigra* and *P. dulcis*, on chromosome 1; and in *P. granatum*, on chromosome 8, where a higher number of *CCO* genes are detected compared to other chromosomes. These results suggest that the genes located on these chromosomes may play a critical role in the species’ adaptation process to stress conditions. In addition, the presence of *CCO* genes on multiple chromosomes in *O. europaea* var. *sylvestris* and *P. nigra* strengthens the likelihood that these species may possess greater genetic diversity and have enhanced mechanisms for adaptation to environmental stresses. The differences in chromosomal distribution across species suggest that the *CCO* gene family may have followed distinct evolutionary pathways. Therefore, following the analysis of the chromosomal distribution of *CCO* genes, a phylogenetic analysis was also conducted.

The phylogenetic analysis conducted on *C. sinensis*, *O. europaea* var. *sylvestris*, *P. nigra*, *P. dulcis*, and *P. granatum* revealed that these genes are classified into three main groups. The number of genes in Group I is lower than in the other groups, and this group is positioned more distantly in the phylogenetic tree. This suggests that it may be the earliest diverging group evolutionarily and potentially have distinct functional roles. Additionally, at least one *CCO* gene from each species (*CsCCO2*, *CsCCO3*, *OeCCO11*, *OeCCO16*, *PnCCO12*, *PnCCO20*, *PdCCO3*, and *PgCCO11*) is present in Group I. This indicates that Group I genes may have been evolutionarily conserved and could share a common biological function across all species. Furthermore, gene structure analysis revealed that all *CCO* genes in this group consist of 6 exons and 5 introns, suggesting they are highly conserved structurally. The conserved motif analysis indicated that OeCCO11 and OeCCO16 proteins have 6 motifs, but the 5th, 6th, 9th, and 10th motifs are missing. Similarly, PnCCO12 and PnCCO20 each contain 6 motifs, with the 3rd, 6th, 9th, and 10th motifs absent. CsCCO3 and PdCCO3 both include 7 motifs, though CsCCO3 lacks the 1st, 3rd, and 9th motifs, and PdCCO3 is missing the 6th, 7th, and 9th motifs. CsCCO2 has 6 motifs, with the 1st, 3rd, 8th, and 9th motifs missing, and PgCCO11 has 5 motifs, lacking the 3rd, 6th, 7th, 9th, and 10th motifs. These motif distributions align with the phylogenetic relationships observed in the analysis. Upon detailed analysis of the phylogenetic tree, it was observed that *PgCCO11* is located in a separate branch from the other *CCO* genes. Similarly, *CsCCO2* was found to be on a different branch from the other genes in Group I. It was concluded that the genes in other branches share similar motifs. The positioning of genes like *PgCCO11* and *CsCCO2* in different branches suggests that these genes may have a more distant evolutionary relationship with the other *CCO* genes. This finding provides valuable insight into how interspecies divergence and genetic diversity have shaped evolutionary accumulation. The other *CCO* genes, on the other hand, share similar motifs, indicating a closer evolutionary relationship. When evaluating Group II and Group III in the phylogenetic tree in detail, 9 *CCO* genes were observed in Group IIA, 13 *CCO* genes in Group IIB, 10 *CCO* genes in Group IIIA, and 45 *CCO* genes in Group IIIB. In terms of gene numbers, the highest number of genes is found in Group IIIB, with *O. europaea* var. *sylvestris* being the species with the most genes in this group. A noteworthy observation is that 14 out of the 23 *CCO* genes in *O. europaea* var. *sylvestris*, 11 out of the 22 *CCO* genes in *P. nigra*, and 12 out of the 17 *CCO* genes in *P. granatum* are located in this group, indicating that the majority of *PgCCO* genes are found in Group IIIB. These differences in gene distribution suggest that Group IIIB genes have been preserved and expanded at varying rates among species throughout phylogenetic processes. The high proportion of this gene group in *O. europaea var. sylvestris*, *P. nigra*, and *P. granatum* suggests that gene duplication events may have played a significant role in these differences. Additionally, the fact that the majority of the identified *CCO* genes in *P. granatum* belong to Group IIIB indicates that the genes in this group may play a more dominant role in specific biological processes. In addition, the genes *PgCCO4*, *PnCCO18*, *PnCCO9*, *PnCCO22*, *OeCCO15*, *PgCCO6*, *PgCCO1*, and *PgCCO8* were observed to distinctly separate from their respective branches in the phylogenetic analysis. The results of gene structure and conserved protein motif analyses further support these findings. Although *PgCCO4* contains five motifs, it has three copies of the seventh motif, which distinguishes it from other *CCO* genes. Moreover, it is the longest *PgCCO* gene and contains a long intron. *PnCCO18*, in contrast to other *PnCCO* genes, contains two long introns. *PnCCO22* consists of a single exon and lacks introns, making it shorter than other *PnCCO* genes. Similarly, the genes *OeCCO15* and *PgCCO1* also contain only a single exon. *PgCCO6* has fewer exons and introns compared to other *PgCCO* genes. *PgCCO8*, which was observed to distinctly separate in the phylogenetic tree, contains only two motifs and is the shortest *PgCCO* gene. In addition, gene structure analysis revealed that some *CCO* genes in the five species studied do not contain introns. The higher presence of these genes in certain species may contribute to faster expression. On the other hand, long introns were detected in some genes; the presence of these long introns may be significant for gene regulation and alternative splicing. The genes *CsCCO16*, *OeCCO8*, *PnCCO18*, *PdCCO7*, and *PgCCO4* were identified as the longest genes in their respective species, and these genes may have a more complex regulatory mechanism in terms of gene expression. In contrast, *CsCCO11*, *OeCCO17*, *PnCCO15*, *PdCCO1*, and *PgCCO8* were observed as the shortest genes, which may provide an advantage for faster transcription. As another analysis, the conserved protein analysis revealed that the differences in motifs found in different plant species may be related to their evolutionary history and strategies for adapting to environmental conditions. For example, the widespread presence of motifs in most proteins of *O. europaea* var. *sylvestris* suggests that this species may have developed a mechanism for resisting environmental stress conditions. Additionally, the fact that *OeCCO17* contains only the 6th motif may indicate that this protein could have a distinct functionality. The presence or absence of motifs may be particularly related to physiological processes such as stress responses or plant development.

One of the analyses conducted in the study is related to elucidating the structural properties of CCO proteins. For this purpose, homology modeling analysis was performed for CsCCO, OeCCO, PnCCO, PdCCO, and PgCCO proteins, and it was determined that all proteins were predicted with 100% reliability. This result supports the high accuracy of the predicted structures. According to the obtained data, β sheets were found to be dominant in all CCO proteins, indicating that β sheets are conserved in CCO proteins. In addition, antiparallel β sheets, β turns, and long loop regions were widely observed in all proteins, further demonstrating the strong structural similarities. Moreover, distinct differences were also identified in the analyses. The absence of α-helices in CsCCO8, CsCCO9, PnCCO8, and PgCCO8 suggests that these proteins may exhibit differences in folding properties. In particular, the lack of both α-helices and transmembrane helices in PgCCO8 indicates that this protein may have a distinct cellular role compared to the others. Indeed, phylogenetic analysis, gene structure analysis, and conserved protein motif analysis also support this distinction for PgCCO8. In contrast, the highest α-helix content (10%) was observed in PgCCO10, suggesting that this protein may possess greater flexibility than the others. Additionally, although transmembrane helices are present at low levels in CsCCO8 and CsCCO9, their presence implies that these proteins are not fully embedded in the membrane but may interact with it, with this interaction potentially being modulated by specific cellular signals. Another notable finding is that the highest β-strand ratio was observed in OeCCO6 and OeCCO13 (47%), followed by OeCCO18 and PnCCO3 (46%). In contrast, the lowest β-strand ratio (29%) was found in PgCCO4. The high β-strand ratio in OeCCO6 and OeCCO13 may indicate stronger substrate binding and interactions within their functional regions. Conversely, the low β-strand ratio in PgCCO4 suggests a more flexible structure, which could indicate increased sensitivity to environmental factors, cellular conditions, or interactions with other molecules.

The subcellular localization analysis of CCO proteins was performed, and these proteins were found in various organelles such as chloroplasts, mitochondria, peroxisomes, the Golgi apparatus, and the nucleus. Differences in the cellular localization of CCO proteins among different plant species suggest that each species may have developed specific adaptation strategies based on its unique biological requirements. These findings may contribute to a better understanding of the evolutionary adaptation mechanisms of plants and their responses to environmental stress conditions. Additionally, the presence of CCO proteins in different organelles indicates their involvement in a wide range of biological processes, from energy production to stress responses. Furthermore, some CCO proteins were found to be localized in more than one organelle. For instance, CsCCO, OeCCO, and PdCCO proteins were detected in the chloroplast and mitochondria, CsCCO proteins in the cytoplasm and mitochondria, OeCCO and PdCCO proteins in the cytoplasm and nucleus, OeCCO proteins in the cytoplasm and endoplasmic reticulum, PnCCO and PdCCO proteins in the endoplasmic reticulum and vacuole or in the cytoplasm and plasma membrane, while PdCCO proteins were found in the mitochondria and plasma membrane. A more specific analysis revealed that certain CCO genes were localized exclusively in specific organelles. CsCCO4, OeCCO3, and PgCCO12 were found only in the chloroplast and mitochondria; CsCCO4 and CsCCO8 only in the cytoplasm and mitochondria; OeCCO13 and PdCCO2 only in the cytoplasm and nucleus; OeCCO17 only in the cytoplasm and endoplasmic reticulum; PnCCO2, PnCCO5, and PdCCO3 only in the endoplasmic reticulum and vacuole; PnCCO18, PdCCO5, and PdCCO6 only in the cytoskeleton and plasma membrane; while PdCCO3 and PdCCO4 were exclusively localized in the mitochondria and plasma membrane. Considering the binary combinations of the mentioned proteins, CsCCO4, OeCCO3, and PgCCO12 are likely involved in energy metabolism and oxidative processes, CsCCO4 and CsCCO8 in the regulation of metabolic intermediates and oxidative stress responses, OeCCO13 and PdCCO2 in cellular signal production and regulation of gene expression, OeCCO17 in protein folding and transport, PnCCO2, PnCCO5, and PdCCO3 in protein synthesis, transport, and cellular waste management, PnCCO18, PdCCO5, and PdCCO6 in response to environmental stresses and intracellular organization, and PdCCO3 and PdCCO4 may play critical roles in both energy production and signal generation in response to environmental stress. The CsCCO proteins were found to localize exclusively to the Golgi for CsCCO6 and to the plasma membrane for CsCCO9, with CsCCO proteins generally concentrated in the chloroplast, mitochondria, and cytosol. This suggests that CsCCO6 may be involved in the secretion or packaging of specific carotenoid derivatives, while CsCCO9 could be linked to intercellular signaling or mechanisms responding to environmental factors. The OeCCO proteins were found to be predominantly localized in the chloroplast and cytosol, with higher concentrations in the mitochondria and nucleus compared to other organelles. This suggests the potential involvement of OeCCO proteins in energy production, gene expression regulation, and oxidative stress responses. In the case of PnCCO proteins, it was determined that 19 out of 22 proteins were localized in the chloroplast, and 16 were in the cytosol. Furthermore, it was found that only PnCCO10 is located in the vacuole. Based on the obtained data, it has been concluded that PnCCO proteins are primarily involved in energy production, stress responses, and photosynthesis processes. The exclusive localization of PnCCO10 in the vacuole suggests that this protein may be associated with the storage of specific metabolites or cellular waste management. In PdCCO proteins, it has been determined that 9 out of 11 proteins are localized in the chloroplast and nucleus, suggesting that these proteins may function in both photosynthesis-related processes and genetic regulation. Additionally, only PdCCO6 was found in the cytoskeleton, and PdCCO4 in the extracellular region. These data suggest that PdCCO6 may be associated with cell shape and internal structural organization, while PdCCO4 could play a role in intercellular communication or defense mechanisms. Regarding PgCCO proteins, it has been determined that PgCCO7 is distributed across various organelles, including the chloroplast, mitochondria, nucleus, Golgi, vacuole, and extracellular region. Similarly, PnCCO12 was found in the chloroplast, mitochondria, cytosol, cytoskeleton, nucleus, plasma, and extracellular regions. The characteristics of PgCCO7 and PnCCO12 suggest that these proteins could affect multiple metabolic processes and act as a link between cellular activities. On the other hand, PgCCO16 has been found to be localized exclusively in the peroxisome, indicating its potential association with the detoxification of reactive oxygen species and lipid metabolism.

As a result of the cis-acting element analysis conducted in this study, a significant diversity of cis-acting elements was identified in the *CCO* genes of the five examined plant species. The gene with the highest number of functionally characterized cis-acting elements was determined to be *PnCCO15* (171), while *OeCCO13* (46) had the lowest. Additionally, *PnCCO15*, *PdCCO1* (165), *PdCCO10* (162), *PgCCO15* (161), and *PnCCO13* (161) were found to contain a higher number of cis-acting elements compared to others, suggesting that these genes may have significant potential in terms of sensitivity to environmental factors and stress response. Although the *CCO* genes exhibited similar motif numbers, the lowest motif count was observed in *CsCCO* (47), while the highest was found in *OeCCO* (54). Motif analysis revealed the presence of motifs associated with low temperature (LTR), drought (MBS), and defense/stress-related TC-rich repeats. This suggests that these genes may contribute to a defense mechanism against environmental stress conditions. Furthermore, the dominance of light-responsive motifs in these genes indicates that *CCO* genes may play an active role in photosynthesis and energy production processes. The TC-rich repeats motif, which is associated with stress, was identified in five copies in the *PnCCO17* gene. Additionally, this gene was found to be intronless and to contain two copies of the 9th motif in the conserved protein motif analysis. miRNA analyses further indicated that *PnCCO17* is associated with the stress response. These findings suggest that *PnCCO17* could be an important genetic target contributing to stress tolerance and highlight it as a potential candidate for further functional studies. Additionally, the presence of the TC-rich repeats motif exclusively in *PdCCO11* among the *PdCCO* genes is noteworthy. This finding suggests that *PdCCO11* may possess a distinct regulatory mechanism related to stress and exhibit a unique function differentiating it from other *PdCCO* genes. Another important finding is the presence of motifs associated with phytohormones. Regulatory motifs such as ABRE (abscisic acid), AuxRR-core (auxin), CGTCA-motif (methyl jasmonate), GARE-motif (gibberellin), TATC-box (gibberellin), TCA-element (salicylic acid), TGA-element (auxin), and TGACG-motif indicate that *CCO* genes interact with phytohormones and that these hormones play a role in regulating plant stress responses. AuxRR-core was identified only in *CsCCO3*, *OeCCO4*, *OeCCO17*, *OeCCO18*, *OeCCO19*, *PnCCO20*, *PdCCO2*, and *PgCCO4*. Motifs such as CGTCA-motif, TCA-element, and TGACG-motif were predominantly detected in *P. nigra*, specifically in the *PnCCO20* gene, while *CsCCO6* and *CsCCO7* contained ABRE and CGTCA-motif more frequently. These findings suggest that *CCO* genes interact with phytohormones and that this interaction may vary at the gene level. In particular, the higher presence of certain motifs in specific genes suggests that these genes may play a more active role in the respective hormone signaling pathways. This provides important insights into the potential functions of *CCO* genes in plant stress response mechanisms. Additionally, some other motifs found in *CsCCO*, *OeCCO*, *PnCCO*, *PdCCO*, and *PgCCO* genes play a significant role in plant developmental processes. For example, motifs associated with anaerobic induction, meristem expression, and flavonoid biosynthesis indicate that these genes are not only involved in stress responses but also in plant growth, development, and metabolic regulation. This suggests that *CCO* genes are not only associated with environmental stress responses but also play a role in fundamental biological processes in plants. Additionally, an important aspect is that the *CsCCO10*, *OeCCO14*, *OeCCO22*, *PnCCO14*, *PnCCO17*, *PdCCO11*, *PgCCO4*, and *PgCCO5* genes contain motifs associated with the regulation of flavonoid biosynthetic genes. This suggests that these *CCO* genes may contribute to the regulation of flavonoid biosynthesis and, consequently, may be associated with the medicinal and pharmacological properties of plants. Given that flavonoids exhibit antioxidant, anti-inflammatory, and antimicrobial activities, the presence of these motifs may indicate a potential association between *CCO* genes and flavonoid-related biological processes. These findings provide a basis for exploring the potential biotechnological applications of *CCO* genes. Specifically, the predicted association of these genes with environmental stress responses and plant development suggests that they may represent promising candidates for future studies aimed at developing more resilient plant species. It should be noted that the cis-regulatory elements identified in this study were predicted exclusively through in silico promoter analysis, which is associated with a relatively high rate of false-positive predictions. Although these predicted motifs may provide valuable insights into the potential regulatory mechanisms of *CCO* genes, their presence alone should not be interpreted as functional evidence of transcriptional regulation or biological activity. Therefore, these predictions should be considered as putative regulatory features that require experimental validation before definitive functional conclusions can be drawn.

In the study, miRNAs targeting *CCO* genes in five different species were identified, and the most and least targeted genes in each species were determined. Among the *CCO* genes, *PdCCO2* (450 miRNAs) was the most targeted, while *OeCCO17* (9 miRNAs) was the least targeted. The remarkably low number of miRNAs targeting *OeCCO17* is noteworthy. In the same species, *OeCCO16* was targeted by 315 miRNAs, whereas none of the stress-related miRNAs analyzed in the present study were associated with *OeCCO17*. Conserved protein motif analysis revealed that OeCCO17 contained only motif 6, and phylogenetic analysis showed that it was positioned in a separate clade. These findings support each other. The observed differences in miRNA targeting among species indicate the evolutionary dynamics of the *CCO* gene family. For instance, the fact that *PdCCO2* is targeted by 450 miRNAs suggests that this gene is under more intense regulatory control compared to those in other species. Similarly, the targeting of *PnCCO1* by 379 miRNAs highlights its potential biological significance. The targeting of *CCO* genes by numerous miRNAs indicates that these genes are susceptible to post-transcriptional regulation and may play crucial roles in plant physiology. Notably, the targeting of these genes by miRNAs known to be associated with environmental stresses supports the hypothesis that *CCO* genes may be involved in stress tolerance. From another perspective, miR170 targets only *PgCCO5*, miR390 targets only PgCCO8, miR851 targets only *CsCCO5* and *PgCCO20*, miR854 targets *only CsCCO3*, *PnCCO1*, *PnCCO18*, *PdCCO2*, *PdCCO11*, and *PgCCO10*, while miR1088 targets only *OeCCO2*. Another important observation is that *PdCCO2*, which is targeted by the highest number of miRNAs, is targeted only by miR156, miR395, miR397, miR408, and miR854. Furthermore, *PgCCO8*, which showed variability in the analyses, also exhibited similar variability in miRNA analysis, being targeted only by miR393, miR396, and miR845. The differences observed in both general analyses and miRNA targeting profiles of *PgCCO8* indicate that this gene has a distinct evolutionary history and likely possesses a specialized biological function compared to other *CCO* genes. Additionally, miR169 (53 *CCO* genes) and miR395 (49 *CCO* genes) have been identified as the miRNAs targeting the most *CCO* genes. This suggests that these miRNAs exert a regulatory effect on the CCO gene family and, consequently, supports the potential roles of CCO genes in abiotic stress responses. The fact that miR169 targets 53 *CCO* genes and miR395 targets 49 *CCO* genes demonstrates the sensitivity of this gene family to environmental signals and its broad regulatory control. The miRNA targeting profiles of the *CCO* genes identified in the present study show direct interaction with several miRNAs that have previously been linked to different abiotic stress responses [54], and the fact that these miRNAs also target the *CCO* genes identified in current study suggests that *CCO* genes may potentially play a role under various abiotic stress conditions. Moreover, miR395, miR397, miR408, miR845, miR854, and miR1088 have been reported to be involved in drought stress responses, with miR395, miR845, and miR854 being highly expressed under drought conditions, while miR397, miR408, and miR1088 are suppressed [55]. The *CCO* genes targeted by miRNAs that are overexpressed under drought stress (miR395, miR845, and miR854) may be downregulated in these stress conditions, thereby contributing to plant adaptation. In contrast, *CCO* genes targeted by suppressed miRNAs (miR397, miR408, and miR1088) may show increased expression under drought stress, directly contributing to stress tolerance. For example, the fact that *OeCCO2* is targeted solely by miR1088 suggests that this gene may have a specific regulatory mechanism related to drought stress. The fact that *PgCCO8* is targeted solely by miR393, miR396, and miR845, and shows variability in general analyses, suggests that this gene may possess a distinct regulatory mechanism compared to other *CCO* genes. Considering that miR845 is overexpressed under drought stress, it is suggested that *PgCCO8* may participate in a drought-specific response mechanism. In conclusion, the relationships between the miRNA targeting profiles of the identified *CCO* genes and stress responses indicate that *CCO* genes may play regulatory roles in abiotic stress responses. This suggests that *CCO* genes may be important not only in metabolic processes but also in environmental adaptation.

The data obtained in the current study show similarities with the results of other studies conducted on *CCO* genes. It has been reported that the majority of CCO proteins in *Citrus clementina* are located in the chloroplast, and that the *CcNCED1* and *CcNCED3* genes are expressed during stress [51]. In *Cucumis sativus*, the proteins are reported to be located in the chloroplast and cytoplasm, and *CCO* genes contain cis-acting elements related to phytohormones, biotic and abiotic stress, and plant development [13]. Similarly, in *Arachis hypogaea*, *CCO* genes have been reported [12] to be associated with light, drought stress, and hormone responsiveness, and CCO proteins have been found to be located in the cytoplasm and chloroplast. Another study [18] reports that in *Litchi chinensis*, similar to the current study, the cis-acting elements most strongly associated with light response were identified, along with cis-acting elements related to phytohormones, plant development, and stress. Additionally, gene expression analysis under drought stress in *Helianthus annuus* revealed significant up-regulation of *HaNCED16* and *HaNCED19* genes, and analysis of *CCO* genes indicated that these genes contain cis-regulatory elements associated with biotic and abiotic stress response, as well as plant growth and development [15]. Moreover, *OsNCED6* and *OsNCED10* genes in *Oryza sativa* have been shown to be up-regulated under salt stress conditions [2]. Furthermore, in *Gossypium hirsutum*, it has been reported that [14] the *GhNCED3a_A/D* and *GhNCED3c_A/D* genes exhibit a significant response to cold, drought, and salt stress.

Collectively, the integration of phylogenetic relationships, gene structures, conserved protein motifs, predicted three-dimensional structures, subcellular localization, cis-regulatory elements, and miRNA-target interactions provides a comprehensive overview of the evolutionary and functional diversification of the *CCO* gene family across the five plant species. Several genes, including *OeCCO16*, *OeCCO17*, *PdCCO2*, *PnCCO17*, and *PgCCO8*, consistently exhibited distinctive characteristics across multiple analyses, suggesting that they represent promising candidates for future functional studies. Overall, the results indicate that *CCO* genes may play important roles in developmental processes, hormone-mediated signaling, and plant responses to environmental stresses. Nevertheless, experimental validation will be necessary to confirm the biological functions and regulatory mechanisms of these candidate genes.

## 5. Conclusions

The analysis conducted on *C. sinensis*, *O. europaea* var. *sylvestris*, *P. nigra*, *P. dulcis*, and *P. granatum* identified 12, 23, 22, 11, and 17 *CCO* genes, respectively. Most CCO proteins were acidic in nature, β-sheets were the dominant structural elements, and the proteins were predicted to localize to chloroplasts, mitochondria, peroxisomes, the cytoplasm, nucleus, Golgi apparatus, plasma membrane, cytoskeleton, vacuoles, and extracellular spaces. The *CCO* genes were unevenly distributed across chromosomes and were classified into three major phylogenetic groups. Gene structure analysis revealed that some *CCO* genes were intronless, whereas others contained long intron regions. Promoter analysis identified numerous cis-acting elements associated with stress responses, phytohormone regulation, anaerobic induction, meristem expression, circadian control, endosperm expression, differentiation of palisade mesophyll cells, regulation of flavonoid biosynthesis, seed-specific regulation, and cell cycle regulation. In addition, numerous stress-responsive miRNA families were predicted to target *CCO* genes, suggesting that post-transcriptional regulation may contribute to their functional diversity. Several genes, including *OeCCO16*, *OeCCO17*, *PdCCO2*, *PnCCO17*, and *PgCCO8*, exhibited distinctive characteristics across multiple analyses, highlighting them as promising candidates for future functional studies. Overall, these findings provide new insights into the evolution and potential biological functions of the *CCO* gene family and establish a valuable foundation for future studies on stress adaptation and molecular breeding in economically important perennial plant species.

## Data Availability

The datasets generated during and/or analysed during the current study are available from the corresponding author on reasonable request.

## Supplementary Materials

The following supporting information can be downloaded at: https://www.mdpi.com/article/10.3390/genes17080903/s1, Supplementary Table S1: Physical positions and physicochemical properties of the identified CCO proteins; Supplementary Table S2: Cis-regulatory elements identified in the promoter regions of *CCO* genes; Supplementary Table S3: Predicted miRNAs targeting *CCO* genes.
